# Untargeted Metabolomics for Unraveling the Metabolic Changes in Planktonic and Sessile Cells of *Salmonella* Enteritidis ATCC 13076 after Treatment with *Lippia origanoides* Essential Oil

**DOI:** 10.3390/antibiotics12050899

**Published:** 2023-05-12

**Authors:** Yuliany Guillín, Marlon Cáceres, Elena E. Stashenko, William Hidalgo, Claudia Ortiz

**Affiliations:** 1 Escuela de Biología, Universidad Industrial de Santander, Bucaramanga 680002, Colombia; yuliany2208193@correo.uis.edu.co; 2 Escuela de Medicina, Universidad Industrial de Santander, Bucaramanga 680002, Colombia; 3 Center for Chromatography and Mass Spectrometry CROM-MASS, School of Chemistry, Universidad Industrial de Santander, Bucaramanga 680002, Colombia; elena@tucan.uis.edu.co; 4 Escuela de Química, Universidad Industrial de Santander, Bucaramanga 680002, Colombia; 5 Escuela de Microbiología y Bioanálisis, Universidad Industrial de Santander, Bucaramanga 680002, Colombia; ortizc@uis.edu.co

**Keywords:** essential oil, antimicrobial agent, microbial resistance, *Salmonella*, biofilm, planktonic cells, metabolomics

## Abstract

Nontyphoidal *Salmonella* species are one of the main bacterial causes of foodborne diseases, causing a public health problem. In addition, the ability to form biofilms, multiresistance to traditional drugs, and the absence of effective therapies against these microorganisms are some of the principal reasons for the increase in bacterial diseases. In this study, the anti-biofilm activity of twenty essential oils (EOs) on *Salmonella enterica* serovar Enteritidis ATCC 13076 was evaluated, as well as the metabolic changes caused by *Lippia origanoides* thymol chemotype EO (LOT-II) on planktonic and sessile cells. The anti-biofilm effect was evaluated by the crystal violet staining method, and cell viability was evaluated through the XTT method. The effect of EOs was observed by scanning electron microscopy (SEM) analysis. Untargeted metabolomics analyses were conducted to determine the effect of LOT-II EO on the cellular metabolome. LOT-II EO inhibited *S*. Enteritidis biofilm formation by more than 60%, without decreasing metabolic activity. Metabolic profile analysis identified changes in the modulation of metabolites in planktonic and sessile cells after LOT-II EO treatment. These changes showed alterations in different metabolic pathways, mainly in central carbon metabolism and nucleotide and amino acid metabolism. Finally, the possible mechanism of action of *L. origanoides* EO is proposed based on a metabolomics approach. Further studies are required to advance at the molecular level on the cellular targets affected by EOs, which are promising natural products for developing new therapeutic agents against *Salmonella* sp. strains.

## 1. Introduction

*Salmonella enterica* is a Gram-negative, facultative anaerobic, flagellated, rod-shaped bacterium belonging to the Enterobacteriaceae family and the causative agent of nontyphoidal salmonellosis [1]. Infections caused by this microorganism are a serious threat to public health and generate economic losses associated with the costs of prevention and treatment of foodborne diseases [2]. *S. enterica* serovar Enteritidis is one of the most common serotypes responsible for gastroenteritis, along with *S. enterica* serovar Typhimurium. These microorganisms act as etiological agents of intestinal and systemic diseases; the most frequent conditions are bacteremia, asymptomatic chronic carrier state, and localized infection [3]. Additionally, it has been reported that several serovars of *S. enterica*, including Enteritidis, can form biofilms through chemical signals of cellular communication, a feature responsible for 80% of bacterial infections in humans [4,5]. The development of biofilm occurs with the production of extracellular polymers that contain extracellular DNA, polysaccharides, and proteins [6,7]. *S.* Enteritidis produces an extracellular matrix with curli and cellulose as the major components [8]. 

Biofilm formation is well known for its physical and biological properties that confer resistance against antibiotics, contributing to the chronicity of infections and their recurrence, which is one of the main challenges in current antibiotic therapy [9]. Moreover, the antibiotic resistance of bacterial biofilms has been attributed to several factors, including (*i*) extracellular polymeric substances (EPS), which act as a physical and chemical barrier; (*ii*) decreased cellular metabolism, which prevents antibiotics acting on dividing cells from presenting any significant effect on microbial cells; and (*iii*) the negative charge of extracellular DNA, which allows binding to positively charged antibiotics, decreasing their effectiveness, among other effects [10,11].

For this reason, it is essential to promote the search for new antimicrobial compounds to counteract this public health problem. Among different antimicrobial compounds, essential oils (EOs) have emerged as a natural promising alternative to conventional antibiotics because they have a broad spectrum of biological properties, including the prevention of biofilm formation, the inhibition of cell communication mechanisms (anti-quorum sensing), and the inhibition of the cell growth of antibiotic-resistant microorganisms [12,13].

The mechanism of action of EOs depends on their chemical composition, and their biological activity cannot be attributed to a single mechanism but to a cascade of reactions involving the entire bacterial cell [9,14]. It has been proposed that components of EOs target the cell wall and cytoplasmic membrane through different mechanisms, including the modification of fatty acid composition, the loss of membrane potential, an increase in cell permeability, the alteration of ATPases and ATP production, the release of cytoplasmic contents, the inhibition of cell communication mechanisms, and alterations in amino acid biosynthesis [15,16].

Rapid advances in omics technology, such as transcriptomics, proteomics, and metabolomics, have improved the sensitivity of biomolecular analysis for testing hypotheses in the detection of genotype and phenotype changes in living organisms. Primarily, metabolomics is a sensitive and promising tool that allows for the identification and quantification of metabolites and provides important information on functional genomics, metabolic engineering, and cell physiology because metabolic profiling allows for a snapshot capture of cell physiology [14,17].

In the present study, twenty EOs were assessed for their antibiofilm activity against *Salmonella enterica* serovar Enteritidis ATCC 13076. Furthermore, *L. origanoides* thymol chemotype EO was chosen for studying the possible mechanism of action through a chemometric approach based on non-targeted metabolomics.

## 2. Results

### 2.1. Plant Material and Chemical Analysis of Essential Oils 

A total of 81 compounds were identified and quantified (>0.1%) in the EOs, and their relative abundances were grouped by chemical class as sesquiterpene hydrocarbons (43%, SH), followed by monoterpene hydrocarbons (17%, MH), oxygenated monoterpenes (16%, OM), oxygenated sesquiterpenes (16%, OS), and oxygenated compounds (8%, OC) (Table S1) [18]. 

### 2.2. Antibiofilm Activity of EOs

*L. origanoides* EO, thymol-*p*-cymene chemotype (LOTC), and thymol chemotype (LOT II) exhibited the highest antibiofilm activity, inhibiting the formation of biofilm on *S*. Enteritidis above 60% at a concentration of 0.13 mg/mL. On the other hand, LOC and LOT-I EOs inhibited biofilm formation by 58% and 52%, respectively, at a concentration of 0.18 mg/mL, and other EOs showed inhibition of biofilm formation ranging from 15–57% at concentrations of 0.75 and 1.5 mg/mL (Table 1, Figure S1).

### 2.3. Cell Viability on Sessile Cells (Biofilm)

**Figure 1:**
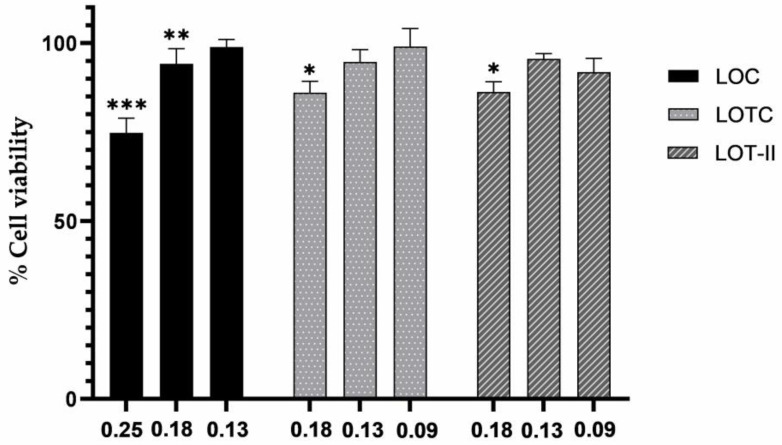
Effect of cell viability of sessile cells of *S.* Enteritidis when treated with different concentrations of essential oils. Data are expressed as mean ± SD (*n* = 3). (* *p* < 0.01, ** *p* < 0.001, *** *p* < 0.0001).

Overall, sessile cells exhibited significant differences in cell viability when treated with LOC EO at concentrations of 0.25 and 0.18 mg/mL. Treatment with LOTC and LOT-II EOs showed significant differences at a concentration of 0.18 mg/mL, while treatment with subinhibitory concentrations generated no significant changes in cell viability. Finally, the percentage of cell viability was higher than 70% in all cases (Figure 1).

### 2.4. Scanning Electron Microscopy (SEM) Analysis

**Figure 2:**
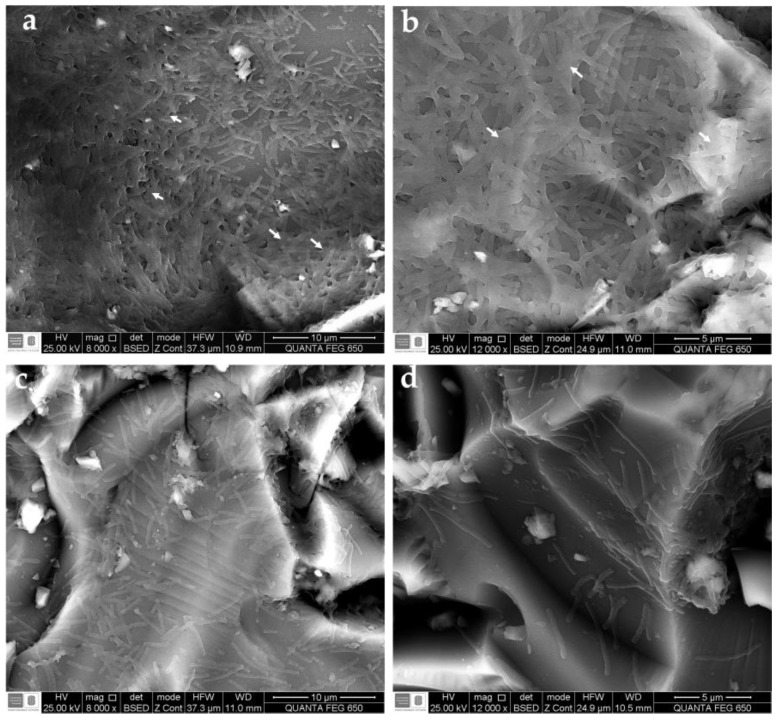
Micrographs of biofilm of *S.* Enteritidis on ground glass coupons obtained by scanning electron microscopy (SEM). (**a**,**b**) Non-treated biofilm of *S*. Enteritidis (control). (**c**,**d**) Biofilm of *S.* Enteritidis treated with subinhibitory concentrations of LOT-II EO. Arrows highlight the areas of the extracellular matrix.

Biofilm cells of *S*. Enteritidis exhibited differences in cell density and bacterial morphology upon treatment with LOT-II EO. Comparing the control and EO-treated cells, differences were easily noticed in the cell density and bacterial morphology, as shown in Figure 2. Micrographs of the biofilm of *S.* Enteritidis (control) showed smooth, bacillary-shaped microorganisms embedded in a dense polysaccharide extracellular matrix, as shown in Figure 2a,b, whereas sessile cells treated with LOT-II EO showed a decrease in the exopolysaccharide matrix and more dispersed cells caused by the anti-biofilm effect of the EO (Figure 2c,d).

### 2.5. Integrity of Cell Membrane Analysis on Planktonic Cells

**Figure 3:**
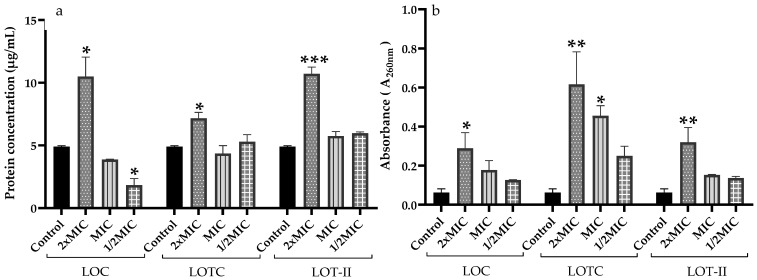
Release of intracellular material from *S.* Enteritidis treated with different EO concentrations. (**a**) Protein release concentration expressed in µg/mL. (**b**) Nucleic acid release measured at 260 nm. Data are expressed as the mean ± SD (*n* = 3). (* *p* < 0.05, ** *p* < 0.01, *** *p* < 0.001).

The cell membrane integrity was determined by measuring the release of biomolecules such as proteins (Figure 3a) and nucleic acids (Figure 3b). The results indicate an increased output of intracellular biomolecules in an EO dose-dependent manner. The concentrations of proteins and nucleic acids in the cell suspensions treated with 2 × MIC presented statistically significant differences when compared to the control.

### 2.6. Metabolomics Data Analysis in Planktonic Cells

**Figure 4:**
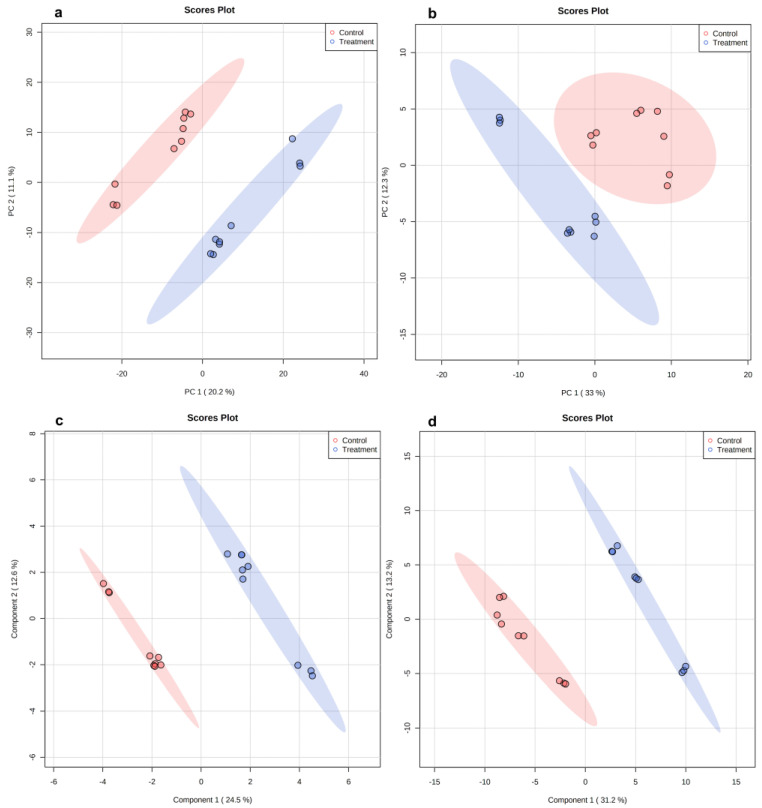
Scoring plots representing PCA and PLS-DA results based on LC/MS data on planktonic cells of *S.* Enteritidis with and without treatment with LOT-II EO. (**a**,**b**) PCA with data obtained from negative and positive ion acquisition modes, respectively. (**c**,**d**) PLS-DA with data obtained from negative and positive ion acquisition modes, respectively. Control (red color) and treated cells (blue color).

Untargeted metabolomics analysis was conducted on planktonic cells to determine the effect of LOT-II EO on the cellular metabolome. LC/MS, operated in the positive (PI) and negative ion (NI) acquisition modes, was used for this purpose. The analyzed data matrix contained 3312 and 5706 features for the NI and PI modes, respectively. Principal component analysis (PCA) plots showed well-defined separation of the untreated (control) and treated (with EO) planktonic cells by both ionization modes (Figure 4). The two principal components (PC1 and PC2) explained 31.3% and 45.3% of the total variability in the data sets for the NI and PI acquisition modes, respectively (Figure 4a,b). According to the results, LOT-II EO induced changes in the metabolome of *S.* Enteritidis. To investigate such changes in more detail, partial least-squares discriminant analysis (PLS-DA) was applied, as shown in Figure 4c,d. The R2 and Q2 parameters showed an adjusted model to high predictability and reliability (0.99 and 0.86, for the NI mode and 0.98 and 0.91 for the PI mode, respectively). Overall, our results demonstrate the significant effect of LOT-II EO on the metabolism of *S.* Enteritidis.

**Figure 5:**
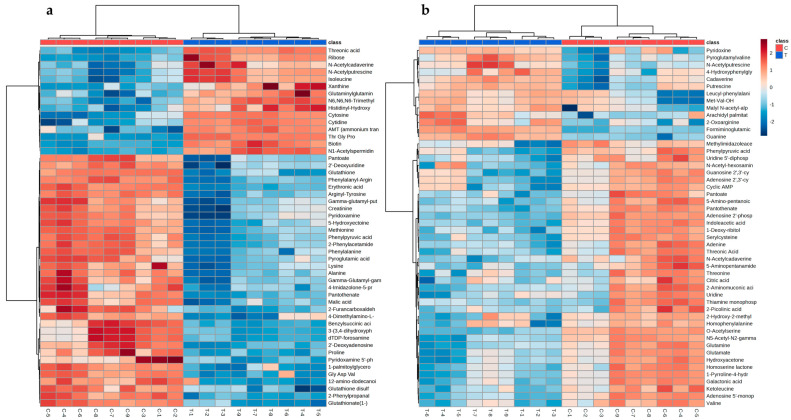
Heatmap plot of the most representative metabolites found to be affected on bacterial cells after treatment with LOT-II EO on (**a**) planktonic and (**b**) sessile cells. Row: metabolites; columns: samples.

According to variable importance in projection (VIP) analysis, a total of 106 putatively annotated compounds (level 2 of metabolite identification, according to the Metabolomics Standards Initiative) were found to be responsible for the grouping separation in PLS-DA analysis, and their identification was performed by a comparison of the mass spectra, fragment ions, and exact mass with those reported in the literature and databases (Table S2) [19,20]. Among the identified differential metabolites, 42 and 64 were found to be up- and downmodulated, respectively. For example, in the NI mode, metabolites such as cytidine, formiminoglutamic acid, 2′,3′-cyclic AMP, and 5-hydroxyisourate were positively modulated in culture cells treated with LOT-II EO, while inosine, guanosine, adenine, 2-oxoglutarate, and thymidine were negatively modulated. For the PI mode, threonic acid, biotin, cytosine, ribose, citric acid, and oxoglutaric acid were in the treated sample, and metabolites such as methionine, lysine, alanine, glutathione, and 4-imidazoline-5-propanoic acid decreased in terms of concentration. The most representative differential metabolites were plotted on a heatmap, allowing for better visualization of the data regarding chemical abundance between the control and the EO-treated bacterial cells (clusters) (Figure 5a).

**Figure 6:**
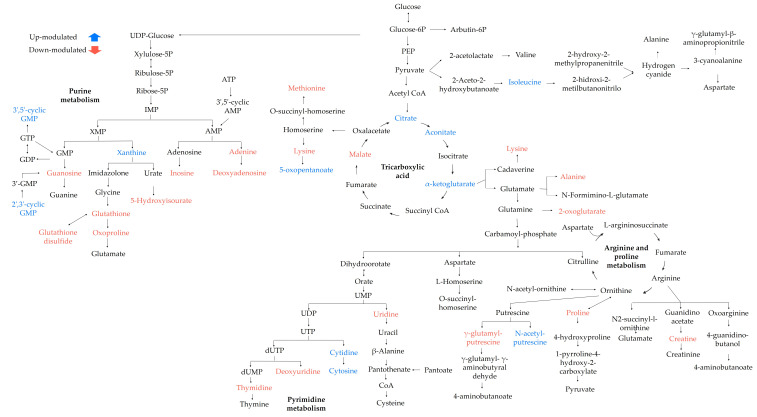
Possible metabolic pathways altered in planktonic cells of *S.* Enteritidis after LOT-II EO treatment. Metabolites colored blue and red indicate up- and downmodulated, respectively, in treated cell samples.

Finally, to understand the changes in the metabolic pathways, an enrichment analysis was performed on the KEGG database. Eight metabolic pathways were possibly affected by the EO treatment in the planktonic cells of *S*. Enteritidis (*p* < 0.05). Table 2 contains the hits referring to differentially modulated metabolites altered in the metabolic pathways, and Figure 6 represents an overview of these metabolic pathways.

### 2.7. Metabolomics Data Analysis of Biofilm Cells

**Figure 7:**
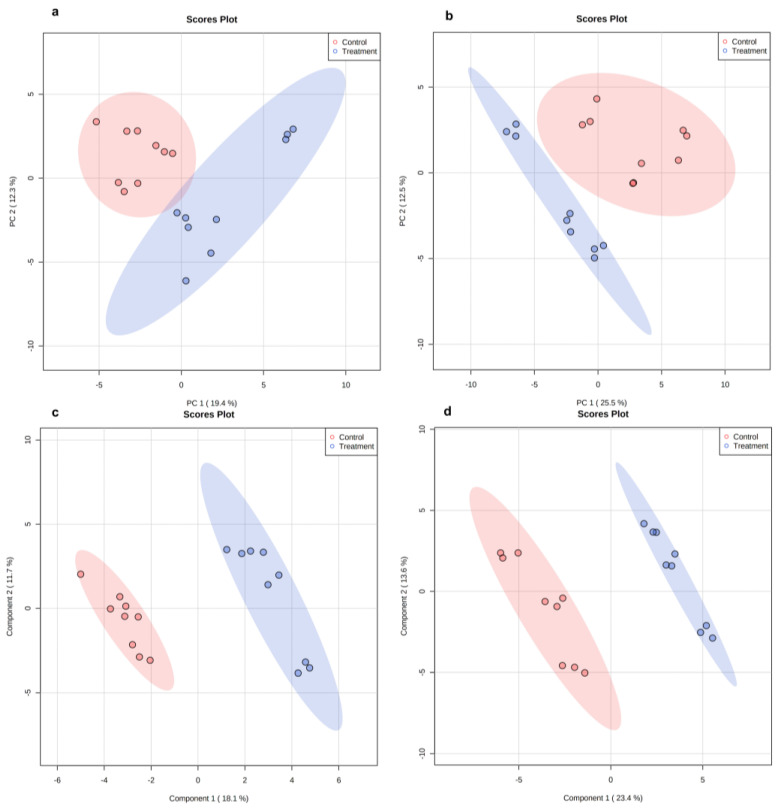
Scoring plots representing PCA and PLS-DA results based on LC/MS data of sessile cells of *S.* Enteritidis cells with and without treatment with LOT-II EO. (**a**,**b**) PCA with data obtained from negative and positive ion acquisition modes, respectively. (**c**,**d**) PLS-DA with data obtained by negative and positive ion acquisition modes, respectively. Control (red color) and treated cells (blue color).

Metabolomics analysis was performed on sessile cells of *S.* Enteritidis in a similar way as described for planktonic cells. For this case, the analyzed data matrix contained 1631 and 3051 features for the NI and PC modes, respectively. PCA plots showed separation between the untreated (control) and treated (with EO) sessile cells in both ionization modes (Figure 7). The two principal components (PC1 and PC2) explained 31.5% and 38% of the total variability for the data sets or the NI and PI acquisition modes, respectively (Figure 7a,b). A supervised method (PLS-DA) was also applied to the data (Figure 7c,d). The values of the R2 and Q2 parameters (0.99 and 0.81 for the NI mode, and 0.99 and 0.89 for the PI mode, respectively) indicate an adjusted model with high predictability and reliability.

As a result, a total of 115 putatively identified metabolites were significantly different (*p* < 0.05) (Table S3). Among the identified metabolites, 30 and 85 were found to be up- and downmodulated, respectively. Metabolites such as formiminoglutamic acid, arbutin 6-phosphate, asparaginyl-isoleucine, malate, guanine, 6-deoxy-glucose, N-acetyl putrescine, cadaverine, putrescine, and arginine were positively modulated in the cultured cells treated with LOT-II EO, while cyclic adenosine monophosphate, cyclic guanosine 2′,3′-phosphate, cyclic acid, inosine, uridine 5′-diphosphate, adenosine 2′-phosphate, glutamate, adenine, cytosine, valine, and uracil were negatively modulated. The most representative differential metabolites were plotted on a heatmap, allowing for better visualization of the data regarding chemical abundance between the control and treated bacterial cells (clusters) (Figure 5b).

**Figure 8:**
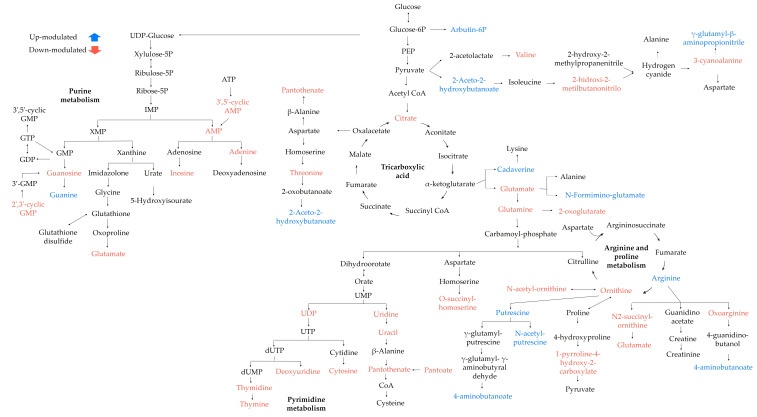
Possible metabolic pathways altered in sessile cells of *S*. Enteritidis after LOT-II EO treatment. Metabolites colored blue and red indicate up- and downmodulated metabolites, respectively, on treated samples.

Finally, to understand the changes in the metabolic pathways, an enrichment analysis was performed on the KEGG database. Twelve metabolic pathways were possibly affected by the EO in the sessile cells of *S.* Enteritidis (*p* < 0.05). Table 3 contains the hits referring to differentially modulated metabolites altered in the metabolic pathways, and Figure 8 represents an overview of these metabolic pathways.

## 3. Discussion

Here, the antibiofilm activity of twenty EOs was explored on *S.* Enteritidis, and a possible mechanism of action of *L. origanoides* EO (LOT-II) is proposed based on a nontargeted metabolomics approach. The chemical composition of EOs depends on several factors, such as geographical origin, soil nutrients, genetic factors, harvesting time, part of the plant, and extraction method [21,22]. This influences the biosynthetic pathways of the secondary metabolites, and therefore, generates different chemical compositions that can differ in antimicrobial properties [23,24,25].

It was found that EOs with the highest biological activity belonged to the *L. origanoides* plant species (Verbenaceae family). Several chemotypes of *L. origanoides* EO were included in the study (Table S1), whose major components corresponded to phenolic monoterpenes, such as thymol and carvacrol, which have been reported in the literature as antimicrobial and antibiofilm agents [26,27,28,29].

The mechanism of action of these compounds has been attributed to their ability to interact with the cell membrane due to their hydrophobic property, which allows them to accumulate, disrupt cellular structures, and cause an increase in permeability, causing leaks of ions and macromolecules, impairment of microbial enzyme systems, and finally, cell death [30,31]. Wang and coworkers tested the alteration in the membrane components of *S. aureus* upon exposure to different concentrations of thymol. When the bacteria were treated with low concentrations, the composition of fatty acids (of the lipid membrane) was greatly affected, mainly in 12-methyltetradecanoic acid and 14-methylhexadecanoic acid, while at higher concentrations of thymol, a disturbance of membrane integrity on *S. aureus*, with reduced cell viability, was observed [24]. Some studies have proven that carvacrol also modifies the fatty acid composition of the cytoplasmic membrane of different microorganisms, such as *E. coli* O157:H7, *S.* Typhimurium, *Pseudomonas fluorescens*, *Brochothrix thermosphacta*, and *S. aureus*, when exposed to sublethal concentrations, increasing the content of unsaturated fatty acids [32].

Moreover, when studying the interaction of thymol with genomic DNA, it was found that thymol can bind to the minor groove of DNA, generating a slight destabilization in the secondary structure of DNA, which hinders the aggregation of DNA molecules [24]. It was also reported that thymol affects the bacterial cell membrane of *S*. Typhimurium, generating a release of important ions for cell metabolism [26]. Regarding the above, our results agree with the previous studies described (Figure 3), because when the bacterial cells were treated with LOT-II EO, an alteration in the membrane permeability of *S.* Enteritidis was evidenced. These results indicate that irreversible damage to cytoplasmic membranes could occur in the bacterial cell, leading to the loss of cellular constituents, such as proteins and some essential molecules, and cell death. The integrity of the cytoplasmic membrane is also a very important factor for cell viability; therefore, the evaluation of extracellular nucleic acids and proteins is an indicator of cell homeostasis since they are responsible for the transfer of information and important cellular and structural functions in bacteria.

Recent studies have not only shown that cell membrane damage causes the cell death of microorganisms, but also that metabolic alterations are generated by EOs [33,34]. In the planktonic cells, our results show that the main differential metabolites corresponded to central carbon metabolism, and amino acid, purine, and pyrimidine metabolism. In addition, aminoacyl-tRNA biosynthesis and glutathione metabolism were also included (Table 2, Figure 6).

Central carbon metabolism is essential for cell growth and the biosynthesis of macromolecules, such as nucleotides, lipids, and proteins. In addition, it is also responsible for the maintenance of cellular homeostasis due to its response to environmental stimuli [35]. This study showed metabolic changes in the tricarboxylic acid cycle (TCA) pathway, where metabolites such as citrate, aconitate, and α-ketoglutarate, were upmodulated, while malate was downmodulated, suggesting a possible interruption in energy metabolism [36]. An accumulation of intermediary metabolites of TCA could indicate that the bacteria would be boosting energy production to maintain cellular homeostasis due to pathways such as amino acid metabolism, which was affected by the EO treatment, e.g., increased *α*-ketoglutarate could not only be related to TCA but also an alternate pathway, such as arginine biosynthesis, alanine, aspartate and glutamate metabolism or amino acid metabolism. On the other hand, reduced levels of malate could reflect a block in the enzyme fumarase, which catalyzes the reversible hydration/dehydration reaction of fumarate to malate; however, to prove this hypothesis, the enzymatic activity of fumarase needs to be investigated under EO treatment [37].

In arginine and proline metabolism, creatinine, gamma-glutamyl-gamma-amino butyraldehyde, proline, and gamma-glutamyl-putrescine were negatively modulated, while N-acetyl putrescine was positively modulated. Proline plays a pivotal role in cell metabolism since it is an important source of carbon and nitrogen and an indispensable osmotic regulator [38,39]. The downmodulation of treated cells would prevent the activation of several stress responses to protect cells from the effects of EO. On the other hand, in alanine, aspartate, and glutamate metabolism, alanine and 2-oxoglutarate metabolites were negatively modulated, similar to other amino acids, such as lysine, phenylalanine, and methionine. Glutamine, alanine, and lysine are major components of peptidoglycan, which is part of the bacterial cell wall, whose function is to protect the internal cellular components and provide support for the cell structure [40]. A decreased concentration of these metabolites, as was observed here, could be associated with an alternative strategy to synthesize peptidoglycan to protect bacterial cells. Methionine is a proteinogenic amino acid of vital importance to initiate protein synthesis and contributes to maintaining the balance of reactive oxygen species. Our results show a decrease in the methionine concentrations, which directly affects protein synthesis but possibly protects the cell against free radicals produced by the effects of EO. Metabolites such as glutathione and glutathione disulfide, related to the oxidative stress response, were also negatively modulated, indicating that the microorganism was trying to counteract the effects generated by LOT-II EO.

In nucleic acid metabolism, the concentrations of 5-hydroxyisourate, guanosine, inosine, adenine, and deoxyadenosine decreased significantly, while the concentrations of guanosine 3′,5′-cyclic monophosphate, guanosine- 2′3′-cyclic monophosphate and xanthine increased in purine metabolism. Purines play an integral role in diverse processes, including energy metabolism, cell signaling, and gene coding [41]. Furthermore, in the metabolism of pyrimidines, thymidine, uridine, and deoxyuridine nucleotides decreased in concentration, while cytidine and cytosine increased significantly. Pyrimidine metabolism is related to repair and survival functions under environmental stress in bacteria [42]. Therefore, altering the metabolic balance of purines and pyrimidines harms the physiological activities of bacteria, such as DNA synthesis and metabolism.

Concerning anti-biofilm activity, the EOs extracted from *L. origanoides* (LOTC) and (LOT-II) inhibited the biofilm formation of *S*. Enteritidis above 60% (Table 1). Similar results were reported by Somrani and coworkers, where clove EO (*Syzygium aromaticum*) inhibited the cell adhesion of *S.* Enteritidis by 49.8% at a concentration of 0.10 mg/mL [43]. Liu and coworkers evaluated the effects of clove and oregano EOs on a *Salmonella* Derby isolate and found significant reductions in biofilm formation of 90.29% and 48.79%, respectively, concluding that EOs inhibited metabolic activity and exopolysaccharide production [44].

One of the possible mechanisms of action of EOs on biofilm blocking has been associated with the inhibition of EPS production, which is secreted by microorganisms during the initial stages of biofilm formation. EPS enhances cell adhesion to the surface and allows for switching from reversible to irreversible attachment of the cells [45]. This mechanism was corroborated in this study by SEM micrographs (Figure 2), where a decrease in the EPS matrix was observed after the EO treatment of *S*. Enteritidis. Other mechanisms of action are related to modifications of gene expression and proteins, which inhibit the production of adhesins and mechanisms of biofilm formation, such as c-di-GMP and quorum sensing. Changes in the modulation of metabolism, which generates alterations in cellular metabolism, have also been documented, in addition to their intrinsic antimicrobial characteristics [46,47,48].

In sessile cells, our results show that the main differential metabolites were involved in amino acid and nucleotide metabolism and pantothenate metabolism (Table 3, Figure 8). Here, changes in the modulation of arginine, glutamate, threonine, valine, and glutamine were found. Glutamic acid, glutamine, and arginine (involved in adhesin formation) are the most representative amino acids in biofilm formation. Glutamate (downmodulated) is one of the key metabolites for energy generation through gluconeogenesis, as well as the starting point for the anaplerosis of nitrogen-containing metabolites. Its function is related to the control of cell division under stress conditions. Glutamine (downmodulated) is a metabolite required for nucleotide and protein biosynthesis, and its function is related to the configuration of biofilm morphology. Arginine (upmodulated) regulates the nitrogen cycle and cellular communication of c-di-GMP [49]. In addition, these metabolites transmit environmental signals for organisms to adapt to different physiological conditions; however, such alterations in the metabolic balance of amino acids affect the formation of biofilms in *S.* Enteritidis.

This study also demonstrated that the exposure of sessile *S.* Enteritidis cells to LOT-II EO generated alterations in central carbon metabolism. The citrate concentration decreased significantly, indicating that the TCA cycle activity was disrupted. Citrate is regenerated in each cycle by the condensation of acetyl-CoA with an oxaloacetate molecule. The reduction in the concentration of this metabolite would affect not only the TCA cycle but also the biosynthesis of fatty acids, which are essential for the biosynthesis of phospholipids in the cell. Another negatively modulated metabolite was pantothenate, an intermediate necessary for TCA metabolism and a key precursor for the synthesis of coenzyme A (related to fatty acid metabolism). In addition, pantothenate regulates the synthesis of glutathione, a metabolite responsible for counteracting apoptosis and cell destruction [42,50].

Nucleotide metabolism was also affected by the EO treatment, and modifications detected in the concentrations of seven metabolites associated with pyrimidine metabolism, cytosine, UDP (uridine diphosphate glucose), uridine, uracil, thymidine, thymine, and glutamine were negatively modulated. The decrease in this metabolic pathway affected the production of curli and cellulose fibers, important components of the bacterial biofilm matrix. Additionally, disruption in pyrimidine biosynthesis can act as a signal of severe nutrient starvation, which in turn can inhibit biofilm formation and promote biofilm dispersion [51]. Purine metabolism was also altered, and eight differentially modulated metabolites were identified, of which glutamine, guanosine, guanosine 2′,3′-cyclic phosphate, inosine, adenine, adenosine 5′-monophosphate, and adenosine 2′,3′-cyclic phosphate decreased in concentration, while guanine was positively modulated. These results evidence changes in the de novo synthesis of adenine from the AMP precursor; this decrease could affect the production of ATP, a fundamental molecule for initial biofilm adhesion [52,53]. Therefore, the disruption of nucleotide metabolism had an adverse effect on the physiological activities of *S.* Enteritidis, such as DNA synthesis and metabolism.

Finally, changes in polyamine metabolism were evidenced, and metabolites such as cadaverine and putrescine were upmodulated, which could indicate that the cells were trying to reduce the production of reactive oxygen species (ROS) generated by the effects of EO, and therefore, prevent damage to biomolecules by oxidative stress [34].

## 4. Materials and Methods

### 4.1. Bacterial Strains and Growth Conditions

*Salmonella enterica* serovar Enteritidis ATCC 13076 (*S*. Enteritidis) strains were obtained from the American Type Culture Collection (ATCC; Rockville, MD, USA). M63 and Mueller Hinton (MH) culture media were obtained from OXOID (Hampshire, UK). All reactions and antibiofilm activity assays were performed using Milli-Q water of 18.2 Ω resistivity, extracted from the Smart 2 Pure Kit (Thermo Fisher Scientific, Waltham, MA, USA). Prior to determining the antibiofilm activity of the EOs, the *Salmonella* strain was grown in M63 medium [54] at 37 °C.

### 4.2. Plant Material and Characterization of Essential Oils

The EOs used in this study were previously reported by [18]. The EOs used were characterized and supplied by the National Center for Research on Agro-Industrialization of Tropical Medicinal Aromatic Plants (CENIVAM), at the Universidad Industrial de Santander (Bucaramanga, Colombia).

Twenty EOs were distilled from the following plants: *Steiractinia aspera* (SA); *Turnera diffusa* (TD-I); *Lippia origanoides*, phellandrene chemotype (LOP); *Calycolpus moritzianus* (CM-I); *Piper aduncum* (PA); *Elaphandra quinquenervis* (EQ); *Hyptis dilatate* (HD); *L. origanoides*, carvacrol chemotype (LOC); *L. origanoides*, β-caryophyllene-thymol chemotype (LOCpT); *L. origanoides*, thymol chemotype (LOT-I); *T. diffusa* (TD-II); *Satureja viminea* (SV); *Psidium sartorianum* (PS); *Varronia curassavica* (VC); *Ocimum basilicum* (OB); *C. moritzianus* (CM-II); *T. diffusa* (TD-III); *L. origanoides*, thymol-p-cymene chemotype (LOTC); *L. origanoides*, thymol chemotype (LOT-II); and *L. micromera* (LM).

The EOs were distilled in Clevengertype equipment adapted to a Samsung MS-1242zk microwave heating system (Seoul, Korea, oven with an output power of 1600 W and 2.4 GHz radiation frequency). The obtained EOs were dried over anhydrous sodium sulfate, weighed, and stored at 4 °C. All hydrodistillations were performed in triplicate [21].

The EOs were analyzed by gas chromatography (Agilent Technologies 6890N Series Network System, Palo Alto, CA, USA) coupled to a mass selective detector (AT, MSD 5975 Inert XL). The EO components were identified based on their mass spectra and linear retention indices (LRI) measured in the two columns—polar and nonpolar—based on the calculated base of the homologous series of *n*-alkanes C_8_–C_25_ and compared with those of different mass spectra, such as WILEY 2008, NIST 2017, and data from the scientific literature [55].

### 4.3. Evaluation of the Antibiofilm Activity of EOs

Biofilm formation was performed in 96-well round-bottom polystyrene microplates. For this determination, an overnight culture of *Salmonella* Enteritidis was grown in 3 mL of M63 medium at 37 °C and 200 rpm. Next, 100 μL of an overnight bacterial culture diluted 1:10 (~1 × 10^6^ colony forming units per milliliter, (CFU/mL) in fresh medium was added to a microplate containing 100 μL of EO. The EOs that exhibited antimicrobial activity were evaluated at subinhibitory concentrations (Table S4). The concentrations evaluated ranged from 0.13 to 1.5 mg/mL. The microplates were incubated at 37 °C for 24 h without shaking. The antibiotic ofloxacin was used as a positive control for microbial inhibition.

The biofilm biomass was quantified by the semiquantitative method of crystal violet staining. The microplate was washed three times with 0.1% (*w*/*v*) aqueous peptone to remove the planktonic cells. Then, 200 μL of 0.4% (*w*/*v*) crystal violet was added to each well for 15 min. Excess crystal violet was removed by three consecutive washes with 0.1% (*w*/*v*) aqueous peptone. Next, 200 μL of 30% (*v*/*v*) acetic acid was added to remove adhering dye, and the absorbance was quantified at 595 nm in an ELISA microplate reader (Bio-Rad, Imark, Hercules, CA, USA) [54].

### 4.4. Metabolic Activity Assay on Biofilm Cells

The 2,3–bis-(2-methoxy-4-nitro-5-sulfophenyl)-5-[(phenylamino)carbonyl]-2H tetrazolium hydroxide (XTT) assay was performed to assess the cellular viability [56]. For this, a 1 mg/mL solution of XTT dissolved in 0.1 M phosphate buffer pH 7, and a solution of menadione at a concentration of 0.4 mM in ethanol was prepared (before each assay). Then, XTT and menadione were mixed in a 20:1 (*v*/*v*) ratio.

After 24 h of biofilm growth, the planktonic cells were removed, and 42 μL of XTT-menadione along with 158 μL of 0.1 M phosphate buffer pH 7 was added to each well of the microplate and incubated at 37 °C for 4 h in the dark. After incubation, the colorimetric change in the solution was measured using an ELISA microplate reader (Bio-Rad, Imark, Hercules, CA, USA) at 490 nm. The intensity of the orange formazan compound, which quantifies the ability of metabolically active sessile cells to reduce tetrazolium salt (XTT), was measured. The percentage of cell viability of the sessile cells was normalized with respect to the untreated cells (control). The data are expressed as the means ± standard deviations for each assay. All experiments were performed in triplicate.

### 4.5. Scanning Electron Microscopy (SEM)

To visualize the effects of the EO on *S.* Enteritidis, scanning electron microscopy (SEM) analysis was performed. The biofilm was formed on 1 × 1.5 cm glass coupons for 24 h at 37 °C. For this, bacterial inoculum diluted 1:10 (~1 × 10^6^ CFU/mL) from an overnight culture was added to a vial containing ground glass coupons, culture medium, and EO at subinhibitory concentrations. The coupons were washed three times with 0.1% (*w*/*v*) aqueous peptone, fixed with 2.5% (*v*/*v*) glutaraldehyde for 60 min, and dehydrated using sequential exposure to isopropanol concentrations ranging from 10 to 95% (*v*/*v*) for 10 min at room temperature [57]. Finally, the morphology of the bacterial cells was observed on a scanning electronic microscope (Quanta 650 FEG electron microscope FEI, Hillsboro, OR, USA), and micrographs were taken on an Everhart Thornley imaging detector ETD detector.

### 4.6. Integrity of the Cell Membrane on Planktonic Cells

*S.* Enteritidis membrane integrity was determined by quantifying the release of cellular constituents according to Diao et al., with slight modifications [58]. Bacterial cells from fresh cultures of the tested microorganisms were washed twice by centrifugation at 4000× *g* for 10 min and resuspended in 0.1 M phosphate buffer pH 7.4. Then, 500 µL of cell suspension was incubated for 24 h at 37 °C in the presence of EOs at three different concentrations (2 × MIC, MIC, ½ MIC). After this time, the samples were centrifuged at 11,000× *g* for 5 min. The supernatant was quantified for nucleic acid and protein concentrations at 260 nm and 280 nm, respectively, in an NP80 nanophotometer (IMPLEN, Munich, Germany). Untreated cells were used as a negative control.

### 4.7. Sample Preparation and Metabolomics Analysis

#### 4.7.1. Extraction of Metabolites from Planktonic and Biofilm Culture Cells

Microbial metabolites were extracted using the method of Zhou et al., with some modifications [59]. The metabolite extraction of planktonic cells was performed at the third hour of microbial growth kinetics, during the exponential phase. In the case of the sessile cells, biofilm formation was performed in microplates containing 1 × 1.5 cm glass coupons. Subsequently, an inoculum of ~1 × 10^6^ CFU/mL, and the selected EOs were added at subinhibitory concentrations. The extraction started with the detachment of the adherent cells after 24 h.

The bacterial cultures were then washed 3 times with 0.1% (*w*/*v*) aqueous peptone and centrifuged at 4000× *g* for 10 min at 4 °C. Then, 500 µL of the extraction solvent, in this case, 50% (*v*/*v*) methanol followed by 500 µL of 50% acetonitrile (ACN), was added. The sample was then sonicated on ice on an ultrasonic homogenizer (ON time = 10, OFF time = 45, and amplitude = 80%), and centrifuged at 13000× *g* for 20 min at 4 °C. The supernatant was deposited in an Eppendorf tube, and the solvent was evaporated using a Savant Speed Vac SPD120 vacuum concentrator (Thermo Fisher Scientific, Waltham, MA, USA). The samples were stored at −80 °C for analysis by UHPLC-ESI-Orbitrap/HRMS. Finally, the residue was reconstituted with 400 µL of 70% (*v*/*v*) methanol aqueous solution containing the internal standards Z-Gly-Tyr-OH and caffeine at a concentration of 5 μM. Quality control (QC) samples were prepared by pooling 20 μL of each cell extract in three QC samples [36,60]. All experiments were carried out with nine biological replicates (*n* = 9).

#### 4.7.2. Analysis of Metabolic Extracts by UHPLC-ESI-Orbitrap/HRMS

The extracted metabolites were analyzed by ultra-high-performance liquid chromatography coupled to high-resolution mass spectrometry (UHPLC-ESI-Orbitrap-HRMS) using OrbitrapTM Exactive Plus equipment (Thermo Fisher Scientific, Sunnyvale, CA, USA) equipped with a Hypersil GOLD TM aQ column (Thermo Fisher Scientific, Sunnyvale, CA, USA; 100 × 2.1 mm, 1.9 μm particle size). The column oven was maintained at 30 °C. The mobile phase, consisting of 0.2% formic acid in water (A) and 0.2% formic acid in acetonitrile (B), was applied for gradient elution using the following program: 100% A was changed linearly to 100% B in 8 min, held constant for 4 min, returned to 100% A in 1 min and held at equilibrium for 3 min. The mobile phase flow rate was 300 μL/min, and the injection volume was 1 μL. Mass spectrometry of the separated metabolites was performed using an Orbitrap-type ion current detection system (Exactive Plus, Thermo Scientific, Sunny, CA, USA), equipped with a heated electrospray ionization interface (HESI). The Orbitrap was operated in full MS scan mode with a resolution of 70,000. The ions were sent for fragmentation to the higher-energy collision dissociation cell (HCD) at different energies (10 eV and 20 eV) in stepped scan mode. For each collision energy, an RFWHM resolution of 35,000 was used, with an AGC of 3 × 10^6^ and a C-trap chamber injection time of 50 ms. All mass spectra were obtained in the *m*/*z* range 50–750 a.m.u. A similar methodology was used for the negative ion acquisition mode.

#### 4.7.3. Data Analysis

The obtained MS raw data were converted into mzXML format files using the MSconvert tool (ProteoWizard 3.0x software http://proteowizard.sourceforge.net/ (accessed on 1 January 2022) and uploaded to the interactive XCMS online platform [61]. The parameter settings for the XCMS data processing were as follows: A multigroup analysis was run in the centWave mode for feature detection (Δppm = 5 ppm, minimum peak width = 5 s, and maximum peak width = 20 s); correction of the retention time was performed with an obiwarp method (profStep = 1); for chromatogram alignment, minfrac = 5, bw = 5, mzwid = 0.025. Tables with the intensities of the detected features were obtained as the output.

The data matrix was filtered according to the coefficient of variation (CV) of the quality control (QC) samples. Data with greater dispersion in the mean were eliminated (CV > 25%). Then, to determine the changes in the metabolic profiles between the control and the EO-treated cells, the data matrix was subjected to uni- and multivariate statistical analysis in Metaboanalyst 5.0 software (https://www.metaboanalyst.ca/ (accessed on 1 September 2022)). [62].

Principal component analysis (PCA) was applied to discriminate groups with different metabolic profiles. To support the results of the PCA, a partial least squares discriminant analysis (PLS-DA) was performed. The most significant features were screened using variable importance in projection (VIP) analysis. Features with a VIP > 1 and a false discovery rate (FDR) < 0.05 were identified using CEU Mass Mediator version 3.0 (http://ceumass.eps.uspceu.es/ (accessed on 1 July 2022)) [63], the Human Metabolome Database https://hmdb.ca/ (accessed on 1 August 2022)), the PubChem (https://pubchem.ncbi.nlm.nih.gov/ (accessed on 1 August 2022)), and MassBank (https://massbank.eu/MassBank/ (accessed on 1 August 2022)). Enrichment and pathway analyses were conducted using MetaboAnalyst 5.0 (https://www.metaboanalyst.ca/ (accessed on 1 September 2022)).

### 4.8. Statistical Analysis

The results were expressed as the mean standard deviation for each of the assays. All experiments were performed in triplicate (*n* = 9 for metabolomics analysis) and one-way analysis of variance (ANOVA) was used to analyze the differences among the treatments. In all cases, the level of significance was 0.05. The assumptions of the normality and equality of variances of the data were previously tested using the Shapiro–Wilk and Levene tests, respectively.

## 5. Conclusions

The EOs distilled from *L. origanoides* species presented the greatest antibiofilm effect on *S.* Enteritidis. The possible mechanism of action of *L. origanoides* thymol chemotype (LOT-II) EO on planktonic cells treated with 2×MIC concentrations was associated with alterations in cell membrane permeability due to the leakage of biomolecules, such as DNA and proteins, while treatment with subinhibitory concentrations generated variations in amino acid metabolism, central carbon metabolism, and nucleic acid metabolism. On the other hand, the effect on biofilm cells treated with subinhibitory concentrations visualized by SEM micrographs evidenced a decrease in EPS production, which is closely related to the alteration in amino acid metabolism. Additionally, LOT-II EO generated changes in purine and pyrimidine metabolism, polyamine metabolism, and pantothenate biosynthesis. These findings provide new insights into the antimicrobial and antibiofilm mechanisms of EOs and promote the study of the pharmacological activity of EOs as natural antibacterial products.

## Figures and Tables

**Table 1:** Antibiofilm activity of EOs on *S.* Enteritidis ATCC 13076. The inhibition percentage was calculated with respect to the control (without EO treatment).

Code	Plant Species	Biofilm Inhibition (%)	EO Concentration (mg/mL)
SA	*Steiractinia aspera* Cuatrec.	57.69 ± 0.18	1.5
TD-I	*Turnera diffusa* Willd	33.27 ± 0.37	1.5
LOP	*Lippia origanoides* H.B.K Phellandrene chemotype	67.40 ± 0.11	1.5
CM-I	*Calycolpus moritzianus* Burret	66.66 ± 0.10	1.5
PA	*Piper aduncum* Lam	-	>1.5
EQ	*Elaphandra quinquenervis* H.Rob	-	>1.5
HD	*Hyptis dilatate* Benth	54.06 ± 0.07	1.5
LOC	*L. origanoides* H.B.K Carvacrol chemotype	58.47 ± 0.08	0.18
LOCpT	*L. origanoides* H.B.K β-Caryophyllene-thymol chemotype	44.00 ± 0.19	0.75
LOT-I	*L. origanoides* H.B.K Thymol chemotype	52.19 ± 0.28	0.18
TD-II	*T. diffusa* Willd	-	>1.5
SV	*Satureja viminea* (L.) Kuntze	-	>1.5
PS	*Psidium sartorianum* (O.Berg) Nied	-	>1.5
VC	*Varronia curassavica* Jacq.	-	>1.5
OB	*Ocimum basilicum* L.	15.25 ± 0.37	1.5
CM-II	*Calycolpus moritzianus* Burret	45.84 ± 0.07	1.5
TD-III	*T. diffusa* Willd	-	>1.5
LOTC	*L. origanoides* H.B.K Thymol-*p*-cymene chemotype	61.14 ± 0.07	0.13
LOT-II	*L. origanoides* H.B.K Thymol chemotype	63.24 ± 0.06	0.13
LM	*L. micromera* Schauer	25.00 ± 0.01	0.75

(-) No inhibition was observed.

**Table 2:** Pathway enrichment analysis with the putatively identified metabolites in planktonic cells.

Pathway Name	Hits	*p*-Value	Identified Metabolites
Arginine and proline metabolism	5/29	4.73 × 10^−3^	Creatinine; γ-glutamyl putrescine; N-acetyl putrescine; proline; γ-glutamyl-γ-aminobutyraldehyde
Citrate cycle (TCA cycle)	4/20	6.79 × 10^−3^	Malate; citric acid; aconitate; 2-oxoglutarate
Aminoacyl-tRNA biosynthesis	6/45	7.30 × 10^−3^	Phenylalanine; methionine; alanine; lysine; isoleucine; proline
Purine metabolism	7/73	2.23 × 10^−2^	5-Hydroxyisourate; 3′,5′-cyclic GMP; guanosine; inosine; adenine; deoxyadenosine; xanthine
Lysine degradation	3/17	2.75 × 10^−2^	Lysine; 5-oxopentanoate; 2-oxoglutarate
Pyrimidine metabolism	5/51	4.85 × 10^−2^	Cytosine; cytidine; deoxyuridine; thymidine; uridine
Alanine, aspartate, and glutamate metabolism	3/22	5.42 × 10^−2^	Alanine; 2-oxoglutaramate; 2-oxoglutarate
Glutathione metabolism	3/22	5.42 × 10^−2^	Glutathione; 5-oxoproline; glutathione disulfide

**Table 3:** Pathway enrichment analysis with the putatively identified metabolites in sessile cells.

Pathway Name	Hits	*p*-Value	Identified Metabolites
Arginine and proline metabolism	9/29	2.52 × 10^−4^	Arginine; putrescine; 4-aminobutanoate; N-acetyl putrescine; ornithine; N2-succinyl-ornithine; glutamate; oxo-arginine; 1-pyrroline-4-hydroxy-2-carboxylate
Arginine biosynthesis	5/16	5.84 × 10^−4^	Arginine; ornithine; N-acetyl ornithine; glutamine; glutamate
Pyrimidine metabolism	7/51	1.96 × 10^−3^	Cytosine; uridine 5′-diphosphate (UDP); uridine; uracil; thymidine; thymine; glutamine
Glutathione metabolism	4/22	1.75 × 10^−2^	Glutamate; ornithine; putrescine; cadaverine
Valine, leucine, and isoleucine biosynthesis	3/22	1.75 × 10^−2^	Threonine; valine; acetyl-2-hydroxy-butanoic acid
Alanine, aspartate, and glutamate metabolism	3/22	1.75 × 10^−2^	2-Oxoglutaramate; glutamate; glutamine
Purine metabolism	8/73	1.83 × 10^−2^	Glutamine; guanosine; guanosine 2′,3′-cyclic phosphate; inosine; adenine; adenosine 5′-monophosphate; adenosine 2′,3′-cyclic phosphate; guanine
β-Alanine metabolism	3/13	2.06 × 10^−2^	Uracil; pantothenate; 4-aminobutanoate
Pantothenate and CoA biosynthesis	4/24	2.37 × 10^−2^	Uracil; valine; pantoate; pantothenate
Glutamine and glutamate metabolism	2/7	3.99 × 10^−2^	Glutamine; glutamate
Cyanoamino acid metabolism	3/17	4.29 × 10^−2^	3-Cyanoalanine; 2-hydroxy-2-methylbutanenitrile; γ-glutamyl-2-aminobutyrate
Aminoacyl-tRNA biosynthesis	5/45	5.73 × 10^−2^	Glutamine; valine; threonine; glutamate; arginine

## Data Availability

The data presented in this study are available upon request from the corresponding author.

## Supplementary Materials

The following supporting information can be downloaded at: https://www.mdpi.com/article/10.3390/antibiotics12050899/s1, Table S1: Major constituents in the EOs studied. The relative amount of each compound is reported as a percentage (%); Figure S1: Anti-biofilm activity of different essential oils on Salmonella enterica serovar Enteritidis ATCC 13076.; Table S2: Putatively identified metabolites altered by the effect of *L. origanoides* LOT-II EO in planktonic cells of *S.* Enteritidis; Table S3: Putatively identified metabolites altered by the effect of LOT-II EO in sessile cells of *S.* Enteritidis; Table S4: Antimicrobial activity of EOs on *Salmonella enterica* serovar Enteritidis ATCC 13076
