# Supplementary material for: Untargeted Metabolomics for Unraveling the Metabolic Changes in Planktonic and Sessile Cells of Salmonella Enteritidis ATCC 13076 after Treatment with Lippia origanoides Essential Oil
Source: Antibiotics (Basel). 2023 May 12;12(5):899. doi: 10.3390/antibiotics12050899 (PMC10215080; doi:10.3390/antibiotics12050899)
Supplement: Supplementary file 1 [file antibiotics-12-00899-s001.zip › antibiotics-2350214-supplementary.pdf]

antibiotics

MDPI

Supplementary Materials

Untargeted Metabolomics for Unraveling the Metabolic Changes in Planktonic and Sessile Cells of *Salmonella* Enteritidis ATCC 13076 after Treatment with *Lippia origanoides* Essential Oil

Yuliany Guillín [1], Marlon Cáceres [2], Elena E. Stashenko [3], William Hidalgo [4,*] and Claudia Ortiz [5]

[1] Escuela de Biología, Universidad Industrial de Santander, Bucaramanga 680002, Colombia
[2] Escuela de Medicina, Universidad Industrial de Santander, Bucaramanga 680002, Colombia
[3] Center for Chromatography and Mass Spectrometry CROM-MASS, School of Chemistry, Universidad Industrial de Santander, Bucaramanga 680002, Colombia
[4] Escuela de Química, Universidad Industrial de Santander, Bucaramanga 680002, Colombia
[5] Escuela de Microbiología y Bioanálisis, Universidad Industrial de Santander, Bucaramanga 680002, Colombia
* Correspondence: whidalgo@uis.edu.co

Table S1. Major constituents in the EOs studied. The relative amount of each compound is reported as a percentage (%).

Code	Plant species	Voucher	Major compounds (>1%)
SA	*Steiractinia aspera* Cuatrec.	UIS Herbarium 20891	α-Pinene (24.9%), β-pinene (14.8%), germacrene D (13.1%), β-phellandrene (10.1%), α-phellandrene (6.3%), sabinene (4.6%), *p*-cymene (4.5%), *trans*-β-caryophyllene (3.1%); α-copaene (2.6%), and limonene (2.4%).
TD-I	*Turnera diffusa* Willd	UIS Herbarium 22037	Dehydrofukinone (25.4%), aristolochene (17.9%), valencene (7.4%), β-selinene (5.2%), *trans*-β-caryophyllene (4.0%), β-elemene (4.0%), premnaspirodiene (3.7%), guaiol (3.5%), germacra-4,5,10-trien-1-α-ol (3.5%), and caryophyllene oxide (3.3%).
LOP	*Lippia origanoides* H.B.K Phellandrene chemotype	COL 560259	*trans*-β-Caryophyllene (18.6%), α-humulene (10.1%), α-phellandrene (9.3%), *p*-cymene (8.7%), 1,8-cineole (6.5%), limonene (4.4%), caryophyllene oxide (3.8%), β-phellandrene (3.1%), camphene (2.5%), and germacrene D (2.2%).
CM-I	*Calycolpus moritzianus* Burret	UIS Herbarium 21982	1,8-Cineole (19.1%), limonene (17.6%), *trans*-β-caryophyllene (6.3%), viridiflorol (5.7%), α-pinene (5.1%), *trans*-geranyl linalool (4.0%), *trans*-nerolidol (3.5%), α-copaene (3.2%), selina-3,7(11)-diene (2.8%), and viridiflorine (2.7%).
PA	*Piper aduncum* L.	COL 587136	Piperitone (14.8%), *trans*-β-caryophyllene (7.4%), viridiflorol (6.5%), limonene (6.0%), δ-cadinene (5.5%), α-pinene (4.6%), α-phellandrene (4.4%), caryophyllene oxide (3.8%), 1,8-cineole (3.6%), and *p*-cymene (3.0%).
EQ	*Elaphandra quinquenervis* H.Rob	COL 587094	Germacrene D (20.7%), α-phellandrene (9.1%), α-pinene (6.8%), *trans*-β-caryophyllene (5.1%), Δ^3-carene (4.9%), limonene (4.5%), β-cubebene (3.5%), α-humulene (2.6%), premnaspirodiene (2.6%), and δ-cadinene (2.6%).
HD	*Hyptis dilatata* Benth	COL 582530	*trans*-β-Caryophyllene (20.2%), camphor (16.1%), Δ3-carene (15.5%), α-pinene (10.5%), palustrol (8.7%), α-gurjunene (4.7%),

Code	Plant species	Voucher	Major compounds (>1%)
			ledol (3.4%), limonene (2.4%), camphene (1.7%), viridiflorine (1.5%), and aromadendrene (1.5%).
LOC	*L. origanoides* H.B.K Carvacrol chemotype	UIS Herbarium 22034	Carvacrol (35%), *p*-cymene (14.4%), thymol (8.0%), γ-terpinene (5.3%), *trans*-β-caryophyllene (4.4%), β-myrcene (2.4%), carvacryl acetate (2.0%), methyl thymyl ether (1.9%), and α-terpinene (1.7%).
LOCpT	*L. origanoides* H.B.K β-Caryophyllene-thymol chemotype	UIS Herbarium 22035	*trans*-β-Caryophyllene (15.1%), thymol (14%), 1,8-cineole (13%), *p*-cymene (12.6%), α-humulene (8.1%), α-phellandrene (7.1%), α-eudesmol (2.6%), caryophyllene oxide (2.5%), γ-terpinene (2.4%), and limonene (2.1%).
LOT-I	*L. origanoides* H.B.K Thymol chemotype	COL 587107	Thymol (75.3%), *trans*-β-caryophyllene (5.4%), carvacrol (4.9%), α-humulene (3.2%), *p*-cymene (2.3%), thymyl acetate (1.6%), methyl thymyl ether (1.3%), caryophyllene oxide (1.3%), and *trans*-β-bergamotene (1.0%).
TD-II	*T. diffusa* Willd	UIS Herbarium 22032	Aristolechene (20.9%), dehydrofukinone (19.3%), valencene (6.5%), β-selinene (5.8%), β-elemene (5.0%), *trans*-β-caryophyllene (4.9%), premnaspirodiene (4.7%), *p*-cymene (3.6%), germacra-4,5,10-trien-1-α-ol (3.6%), and guaiol (3.3%).
SV	*Satureja viminea* (L.) Kuntze	COL 566449	*p*-Menth-3-en-8-ol (32.4%), pulegone (16.1%), *trans*-9-*epi*-caryophyllene (8.9%), *trans*-β-caryophyllene (8.4%), caryophyllene oxide (4.3%), spathulenol (3.6%), benzyl benzoate (2.4%), δ-cadinene (2.2%), *trans*-pulegol (1.8%), and *p*-mentha-3,8-diene (1.5%).
PS	*Psidium sartorianum* (O.Berg) Nied	COL 578359	*trans*-β-Caryophyllene (12.7%), caryophyllene oxide (12.0%), dehydrofukinone (7.5%), caryophylla-4(12),8(13)-dien-5-β-ol (4.8%), germacrene B (4.1%), 1,8-cineole (3.7%), *p*-cymene (2.9%), β-pinene (2.7%), selina-3,7(11)-diene (2.5%), β-selinene (2.1%), and premnaspirodiene (2.0%).
VC	*Varronia curassavica* Jacq.	COL 559446	*trans*-β-Caryophyllene (19.2%), germacrene D (12.3%), *trans*-β-guaiene (11.8%), α-pinene (9.4%), α-copaene (7.0%), β-pinene (4.1%), bicyclogermacrene (3.9%), β-elemene (2.8%), δ-cadinene (2.8%), and α-humulene (2.7%).
OB	*Ocimum basilicum* L.	UIS Herbarium 22227	Linalool (42.7%), estragole (18.6%), 1,8-cineole (8.1%), germacrene D (4.9%), *epi*-α-cadinol (4.2%), γ-cadinene (3.7%), α-humulene (2.5%), β-elemene (2.2%), bicyclogermacrene (2.2%), and *trans*-α-bergamotene (1.1%).
CM-II	*C. moritzianus* Burret	UIS Herbarium 21982	1,8-Cineole (15.4%), limonene (14.7%), viridiflorol (7.1%), *trans*-geranyllinalool (6.7%), *trans*-β-caryophyllene (6.2%), β-selinene (5.8%), *trans*-nerolidol (4.0%), α-pinene (3.5%), selina-3,7(11)-diene (3.0%), and α-copaene (3.0%).
TD-III	*T. diffusa* Willd	Herbarium UIS 22037	Aristolochene (20.6%), dehydrofukinone (17.3%), *p*-cymene (5.8%), β-selinene (5.6%), valencene (5.2%), premnaspirodiene (4.2%), caryophyllene oxide (3.6%), *trans*-β-caryophyllene (2.8%), germacra-4,5,10-trien-1α-ol (2.4%).
LOTC	*L. origanoides* H.B.K Thymol- *p*-cymene chemotype	Herbarium UIS 22039	Thymol (49.4%), *p*-cymene (19.1%), γ-terpinene (9.2%), β-myrcene (5.2%), α-terpinene (2.9%), carvacrol (2.7%), methyl thymyl ether (1.8%), *trans*-β-caryophyllene (1.6%), *cis*-β-ocimene (1.2%), and limonene (0.9%).
LOT-II	*L. origanoides* H.B.K Thymol chemotype	Herbarium UIS 22036	Thymol (71.7%), *p*-cymene (10.5%), carvacrol (4.4%), β-myrcene (2.1%), γ-terpinene (2.0%), caryophyllene oxide (1.6%), methyl

Code	Plant species	Voucher	Major compounds (>1%)
			thymyl ether (0.9%), *trans*-β-caryophyllene (0.9%), humulene epoxide II (0.7%), and terpinen-4-ol (0.7%).
LM	*L. micromera* Schauer	COL 560986	*p*-Cymene (26.8%), methyl thymyl ether (26.3%), thymol (17.8%), thymyl acetate (5.7%), γ-terpinene (5.4%), 1,8-cineole (5.1%), α-terpinene (2.0%), β-myrcene (2.0%), *trans*-β-caryophyllene (1.7%), α-thujene (1.3%), and caryophyllene oxide (0.9%).

Table S2. Putatively identified metabolites altered by the effect of *L. origanoides* LOT-II EO in planktonic cells of *S.* Enteritidis.

Metabolite	Formula	Adduct	*m/z*	Δppm	RT	Modulated	Ionization mode
Glutathione disulfide	$C_{20}H_{32}N_6O_{12}S_2$	$[M-H]^-$	611.1434477	1.11	1.08	DOWN	ESI(−)
Glutathione	$C_{10}H_{17}N_3O_6S$	$[M-H]^-$	306.076014	0.11	1.16	DOWN	ESI(−)
Cytidine	$C_9H_{13}N_3O_5$	$[M-H]^-$	242.0775838	0.43	0.85	UP	ESI(−)
2'-Deoxyuridine	$C_9H_{12}N_2O_5$	$[M-H]^-$	227.0665	1.27	2.04	DOWN	ESI(−)
2,5-Dioxopentanoate	$C_5H_6O_4$	$[M-H]^-$	129.0181634	4.77	0.49	UP	ESI(−)
Gly Asp Val	$C_{11}H_{19}N_3O_6$	$[M-H]^-$	288.1196335	0.25	0.96	DOWN	ESI(−)
2',3'-Cyclic AMP	$C_{10}H_{12}N_5O_6P$	$[M-H]^-$	328.0447135	0.07	2.00	UP	ESI(−)
Thymidine	$C_{10}H_{14}N_2O_5$	$[M-H]^-$	241.0823454	0.39	2.78	DOWN	ESI(−)
Pantoate	$C_6H_{12}O_4$	$[(M-H)-H_2O]^-$	129.0545647	4.61	3.06	DOWN	ESI(−)
Malic acid	$C_4H_6O_5$	$[(M-H)-H_2O]^-$	115.0024718	5.72	0.49	DOWN	ESI(−)
Serylcysteine	$C_6H_{12}N_2O_4S$	$[(M-H)-H_2O]^-$	189.0329752	2.14	0.53	UP	ESI(−)
Cys Thr Cys	$C_{10}H_{19}N_3O_5S_2$	$[(M-H)-H_2O]^-$	306.0573702	2.77	1.16	DOWN	ESI(−)
Uridine	$C_9H_{12}N_2O_6$	$[M-H]^-$	243.0616018	0.44	1.36	DOWN	ESI(−)
O-Succinyl-homoserine	$C_8H_{13}NO_6$	$[(M-H)-H_2O]^-$	200.055546	1.71	1.16	DOWN	ESI(−)
Gln-Pro	$C_{10}H_{17}N_3O_4$	$[M-H]^-$	242.1139328	0.60	0.93	UP	ESI(−)
2-oxoglutaramate	$C_5H_7NO_4$	$[2M-H]^-$	289.0672693	0.30	1.36	DOWN	ESI(−)
Pro Gly Gln	$C_{12}H_{20}N_4O_5$	$[M-H]^-$	299.1355365	0.01	0.53	DOWN	ESI(−)
3'-Keto-3'-deoxy-AMP	$C_{10}H_1N_5O_7P$	$[M-H]^-$	344.0394785	0.35	1.80	UP	ESI(−)
3-Hydroxy-2-methylpyridine-5-carboxylate	$C_7H_7NO_3$	$[M-H]^-$	152.0341913	3.74	1.40	DOWN	ESI(−)
Adenine	$C_5H_5N_5$	$[M-H]^-$	134.0460744	444	0.81	DOWN	ESI(−)
Formiminoglutamic acid	$C_6H_{10}N_2O_4$	$[(M-H)-H_2O]^-$	155.045069	3.81	0.73	UP	ESI(−)
Asn Leu Asp	$C_{14}H_{24}N_4O_7$	$[M-H]^-$	359.1566792	0.02	2.87	DOWN	ESI(−)
Methionine	$C_5H_{11}NO_2S$	$[M-H]^-$	148.0425725	4.37	0.69	DOWN	ESI(−)
N-(3-Oxohexanoyl)homoserine lactone	$C_{11}H_{17}NO_3S$	$[M-H]^-$	242.0856159	2.21	2.08	DOWN	ESI(−)
Asp Lys Pro	$C_{15}H_{26}N_4O_6$	$[M-H]^-$	357.1774182	0.05	3.07	DOWN	ESI(−)
Leu His Val	$C_{21}H_{33}N_3O_4$	[(M+Na)-2H]-	412.2194875	4.20	3.55	UP	ESI(−)
2-Hydroxyethanesulfonate	$C_2H_6O_4S$	$[M-H]^-$	124.9902573	4.74	0.51	DOWN	ESI(−)
Threonate	$C_4H_8O_5$	$[M-H]^-$	135.0287462	4.39	0.65	DOWN	ESI(−)
Erythronic acid	$C_4H_8O_5$	$[(M-H)-H_2O]^-$	117.0181309	5.54	1.08	DOWN	ESI(−)
3-Phosphoadenylylselenate	$C_{10}H_{15}N_5O_{13}P_2Se$	$[M-3H]^-$	183.9692942	1.23	0.85	UP	ESI(−)
Glutamylglycine	$C_7H_{12}N_2O_5$	$[(M-H)-H_2O]^-$	185.0557955	2.34	0.77	DOWN	ESI(−)

Val-Asn	$C_9H_{17}N_3O_4$	$[M-H]^-$	230.1139313	0.64	1.36	DOWN	ESI(−)
2-(2-Carboxy-4-methylthiazol-5-yl)ethyl phosphate	$C_7H_{10}NO_6PS$	$[M-H]^-$	265.9883458	1.74	1.11	DOWN	ESI(−)
Asn Leu Asp	$C_{14}H_{24}N_4O_7$	$[(M-H)-H_2O]^-$	341.1460239	0.22	0.53	DOWN	ESI(−)
Leucinic acid	$C_6H_{12}O_3$	$[M-H]^-$	131.0702067	4.60	4.3	DOWN	ESI(−)
Inosine	$C_{10}H_{12}N_4O_5$	$[M-H]^-$	267.0729551	0.05	2.35	DOWN	ESI(−)
Guanosine	$C_{10}H_{13}N_5O_5$	$[M-H]^-$	282.0838703	0.10	2.40	DOWN	ESI(−)
gamma-Glutamylvaline	$C_{10}H_{18}N_2O_5$	$[M-H]^-$	245.1135962	0.58	1.32	DOWN	ESI(−)
Glutaminylleucine	$C_{11}H_{21}N_3O_4$	$[M-H]^-$	258.1454303	0.19	2.87	UP	ESI(−)
5-Ureido-4-imidazole carboxylate	$C_5H_6N_4O_3$	$[(M-H)-H_2O]^-$	151.0249536	4.28	1.12	UP	ESI(−)
Hydroxypropanedioic acid	$C_3H_4O_5$	$[M-H]^-$	118.9974038	5.34	1.76	DOWN	ESI(−)
3,4-Dihydroxymandelic acid	$C_8H_8O_5$	$[(M-H)-H_2O]^-$	165.0182595	3.15	3.53	DOWN	ESI(−)
Phenylalanine	$C_9H_{11}NO_2$	$[M-H]^-$	164.0706391	3.11	2.44	DOWN	ESI(−)
5-Hydroxyisourate	$C_5H_4N_4O_4$	$[(M-H)-H_2O]^-$	165.0043526	3.07	0.88	UP	ESI(−)
Thr Gly Pro	$C_{11}H_{19}N_3O_5$	$[(M+H)-H_2O]^+$	256.1287395	3.86	0.50	UP	ESI (+)
N1-Acetylspermidine	$C_9H_{21}N_3O$	$[M+H]^+$	188.1756703	3.24	0.47	UP	ESI (+)
12-amino-dodecanoic acid	$C_{12}H_{25}NO_2$	$[(M+H)-H_2O]^+$	198.1851465	3.19	5.55	DOWN	ESI (+)
N-Acetylputrescine	$C_6H_{14}N_2O$	$[M+H]^+$	131.1180264	3.07	0.51	UP	ESI (+)
Biotin	$C_{10}H_{16}N_2O_3S$	$[M+H]^+$	245.0958319	0.60	0.82	UP	ESI (+)
Cytosine	$C_4H_5N_3O$	$[M+H]^+$	112.0508077	2.43	0.51	UP	ESI (+)
Isoleucine	$C_6H_{13}NO_2$	$[(M+H)-H_2O]^+$	114.0915482	2.90	0.51	UP	ESI (+)
Glutathione	$C_{10}H_{17}N_3O_6S$	$[M+H]^+$	308.090624	3.26	0.54	DOWN	ESI (+)
2-Phenylpropanal	$C_9H_{10}O$	$[2M+Na]^+$	291.1376434	5.33	0.96	DOWN	ESI (+)
2-Hydroxy-2-ethylsuccinic acid	$C_6H_{10}O_5$	$[M+H]^+$	163.0613814	4.54	1.17	UP	ESI (+)
12-amino-dodecanoic acid	$C_{12}H_{25}NO_2$	$[M+H]^+$	216.1957101	2.95	5.56	DOWN	ESI (+)
Ribose	$C_5H_{10}O_5$	$[(M+H)- H_2O]^+$	133.0495374	4.07	0.51	UP	ESI (+)
Pantothenate	$C_9H_{17}NO_5$	$[M+H]^+$	220.1178505	2.90	2.94	DOWN	ESI (+)
1-palmitoylglycerophosphocholine	$C_{24}H_{51}NO_7P$	$[M+2Na]^+$	271.1590589	3.19	0.54	DOWN	ESI (+)
N6,N6,N6-Trimethyl-lysine	$C_9H_{20}N_2O_2$	$[(M+H)-H_2O]^+$	171.1491314	3.49	0.47	UP	ESI (+)
Histidinyl-Hydroxyproline	$C_{11}H_{16}N_4O_4$	$[M+H]^+$	269.1240566	3.43	0.78	UP	ESI (+)
Citric acid	$C_6H_8O_7$	$[(M+H)-H_2O]^+$	175.0235655	3.96	0.77	UP	ESI (+)
Cytidine	$C_9H_{13}N_3O_5$	$[M+H]^+$	244.092565	3.17	0.82	UP	ESI (+)
Glutaminylglutamine	$C_{10}H_{18}N_4O_5$	$[M+H]^+$	275.1346363	3.28	1.05	UP	ESI (+)
Glycerol	$C_3H_8O_3$	$[M+Na]^+$	115.0369811	1.12	0.86	UP	ESI (+)
Acetyl tributyl citrate	$C_{20}H_{34}O_8$	$[M+H]^+$	403.2320393	2.85	8.64	UP	ESI (+)
Arbutin	$C_{12}H_{16}O_7$	$[M+H]^+$	273.0965514	3.18	8.63	UP	ESI (+)
N-Acetylcadaverine	$C_7H_{16}N_2O$	$[M+H]^+$	145.1335297	3.79	0.88	UP	ESI (+)
N-Cyclohexylformamide	$C_7H_{13}NO$	$[M+H]^+$	128.1070842	3.47	6.19	UP	ESI (+)
5-Hydroxykynurenine	$C_{10}H_{12}N_2O_4$	$[M+2H]^+$	113.0477629	0.77	0.82	UP	ESI (+)
Arg Val Ser	$C_{14}H_{28}N_6O_5$	$[M+H]^+$	361.2215333	4.41	8.64	UP	ESI (+)

4-Dimethylamino-phenyl-alanine	$C_{11}H_{16}N_2O_2$	$[M+H]^+$	209.1282274	3.69	0.51	DOWN	ESI (+)
Benzylsuccinic acid	$C_{11}H_{12}O_4$	$[M+H]^+$	209.0807856	2.84	6.70	DOWN	ESI (+)
1-(beta-Ribofuranosyl)-1,4-dihydronicotinamide	$C_{11}H_{16}N_2O_5$	$[M+H]^+$	257.1127145	3.98	2.32	DOWN	ESI (+)
Ser Arg Tyr	$C_{18}H_{28}N_6O_6$	$[M+H]^+$	425.2138845	2.27	8.64	UP	ESI (+)
Oxoglutaric acid	$C_5H_6O_5$	$[(M+H)-H_2O]^+$	129.0183033	3.69	8.65	UP	ESI (+)
Xanthine	$C_5H_4N_4O_2$	$[M+H]^+$	153.0406565	3.87	1.13	UP	ESI (+)
2-Furancarboxaldehyde	$C_5H_4O_2$	$[M+H]^+$	97.02875159	2.04	0.47	DOWN	ESI (+)
3-(3,4-dihydroxy-phenyl)prop-2-enoic acid	$C_9H_8O_4$	$[M+H]^+$	181.0494479	3.49	6.70	DOWN	ESI (+)
dTDP-forosamine	$C_{18}H_{31}N_3O_{12}P_2$	$[M+3H]^+$	182.0528667	5.76	6.70	DOWN	ESI (+)
Adenine	$C_5H_5N_5$	$[M+H]^+$	136.0617373	4.28	3.70	DOWN	ESI (+)
Arginyl-Tyrosine	$C_{15}H_{23}N_5O_4$	$[M+H]^+$	338.1818176	2.96	1.09	DOWN	ESI (+)
5-Hydroxyectoine	$C_6H_{10}N_2O_3$	$[M+H]^+$	159.0763844	3.61	0.49	DOWN	ESI (+)
Gentisate aldehyde	$C_7H_6O_3$	$[(M+H)-H_2O]^+$	121.0286007	2.88	9.87	DOWN	ESI (+)
Phenylalanyl-Arginine	$C_{15}H_{23}N_5O_3$	$[M+H]^+$	322.1868297	3.35	2.28	DOWN	ESI (+)
4-Acetamido-2-aminobutanoic acid	$C_6H_{12}N_2O_3$	$[M+H]^+$	161.0920366	3.55	0.51	UP	ESI (+)
5-Hydroxykynurenine	$C_{10}H_{12}N_2O_4$	$[M+2H]^+$	113.0478575	1.65	0.51	DOWN	ESI (+)
Methionine	$C_5H_{11}NO_2S$	$[M+H]^+$	150.0582532	4.11	0.70	DOWN	ESI (+)
Aconitic acid	$C_6H_6O_6$	$[(M+H)-H_2O]^+$	139.0025748	3.99	8.64	UP	ESI (+)
Histamine	$C_5H_9N_3$	$[M+H]^+$	112.0871771	2.61	0.46	UP	ESI (+)
5-oxo-pentanoic acid	$C_5H_8O_3$	$[M+H]^+$	117.0548172	2.92	0.50	UP	ESI (+)
Pyridoxamine 5'-phosphate	$C_8H_{13}N_2O_5P$	$[M+2H]^+$	125.0365667	5.17	0.51	DOWN	ESI (+)
gamma-Glutamyl-gamma-aminobutyraldehyde	$C_9H_{16}N_2O_4$	$[M+H]^+$	217.1181573	3.09	4.60	DOWN	ESI (+)
Gamma-glutamyl-putrescine	$C_9H_{19}N_3O_3$	$[(M+H)-H_2O]^+$	200.1392363	3.31	0.70	DOWN	ESI (+)
Pyroglutamic acid	$C_5H_7NO_3$	$[M+H]^+$	130.0499772	3.32	0.82	DOWN	ESI (+)
Urocanic acid	$C_6H_6N_2O_2$	$[(M+H)-H_2O]^+$	121.0398063	3.08	1.04	UP	ESI (+)
Cyclic GMP	$C_{10}H_{12}N_5O_7P$	$[M+H]^+$	346.0542684	2.83	1.77	UP	ESI (+)
2-Phenylacetamide	C_8H_9NO	$[M+H]^+$	136.0757	3.89	3.77	DOWN	ESI (+)
4-Imidazolone-5-propanoic acid	$C_6H_8N_2O_3$	$[M+H]^+$	157.0607652	3.46	0.50	DOWN	ESI (+)
Lysine	$C_6H_{14}N_2O_2$	$[(M+H)-H_2O]^+$	129.1023572	3.27	0.50	DOWN	ESI (+)
Proline	$C_5H_9NO_2$	$[M+H]^+$	116.0707922	3.08	0.51	DOWN	ESI (+)
Alanine	$C_3H_7NO_2$	$[M+H]^+$	90.05536955	1.44	0.47	DOWN	ESI (+)
Phenylpyruvic acid	$C_9H_8O_3$	$[M+H]^+$	165.0545978	3.40	1.09	DOWN	ESI (+)
Pyridoxamine	$C_8H_{12}N_2O_2$	$[M+H]^+$	169.0970723	3.71	2.71	DOWN	ESI (+)
2'-Deoxyadenosine	$C_{10}H_{13}N_5O_3$	$[M+H]^+$	252.1089583	2.78	2.57	DOWN	ESI (+)
Creatinine	$C_4H_7N_3O$	$[M+H]^+$	114.066471	2.27	0.50	DOWN	ESI (+)

*RT: retention time.

Table S3. Putatively identified metabolites altered by the effect of LOT-II EO in sessile cells of *S.* Enteritidis.

**Metabolite**	**Formula**	**Adduct**	***m/z***	**Δppm**	**RT**	**Modulated**	**Ionization mode**
Formiminoglutamic acid	$C_6H_{10}N_2O_4$	$[(M-H)-H_2O]^-$	155.04506	3.81	0.54	UP	ESI(−)

Cyclic AMP	$C_{10}H_{12}N_5O_6P$	[M-H]⁻	328.04478	0.29	1.69	DOWN	ESI(−)
N-(3,4-Dichlorophenyl)-malonamate	$C_9H_7Cl_2NO_3$	[(M+Na)-2H]⁻	267.9544	0.09	0.78	DOWN	ESI(−)
Guanosine 2',3'-cyclic phosphate	$C_{10}H_{12}N_5O_7P$	[M-H]⁻	344.0395	0.08	1.78	DOWN	ESI(−)
Serylcysteine	$C_6H_{12}N_2O_4S$	[(M-H)-H2O]⁻	189.03301	1.95	0.51	DOWN	ESI(−)
Arachidyl palmitate	$C_{36}H_{72}O_2$	[M-3H]⁻	177.84367	2.47	0.42	UP	ESI(−)
Adenosine 5′-monophosphate	$C_{10}H_{14}N_5O_7P$	[M-H]⁻	346.05517	0.21	0.50	DOWN	ESI(−)
1-Deoxy-ribitol	$C_5H_{12}O_4$	[(M-H)-H2O]⁻	117.05445	0.44	2.37	DOWN	ESI(−)
Arbutin 6-phosphate	$C_{12}H_{17}O_{10}P$	[(M+HCOOH)-H]⁻	397.0536	0.10	5.37	UP	ESI(−)
(1,2-Dichlorovinyl)glutathione	$C_{12}H_{17}Cl_2N_3O_6S$	[(M+Na)-2H]⁻	421.99864	2.27	5.38	UP	ESI(−)
1-Pyrroline-4-hydroxy-2-carboxylate	$C_5H_7NO_3$	[M-H]⁻	128.0341	4.48	0.43	DOWN	ESI(−)
Galactonic acid	$C_6H_{12}O_7$	[M-H]⁻	195.0501	1.65	0.47	DOWN	ESI(−)
Aspartyl-Proline	$C_9H_{14}N_2O_5$	[M-H]⁻	229.08219	1.06	1.33	DOWN	ESI(−)
2-Oxoarginine	$C_6H_{11}N_3O_3$	[M-H]⁻	172.07164	3.30	0.47	DOWN	ESI(−)
2-Keto-glutaramic acid	$C_5H_7NO_4$	[2M-H]⁻	289.06724	0.30	1.37	DOWN	ESI(−)
Ketoleucine	$C_6H_{10}O_3$	[(M-H)-H2O]⁻	129.05456	4.61	7.69	DOWN	ESI(−)
O-Succinyl-homoserine	$C_8H_{13}NO_6$	[(M-H)-H2O]⁻	200.05548	1.71	0.47	DOWN	ESI(−)
Uridine	$C_9H_{12}N_2O_6$	[M-H]⁻	243.06162	0.44	1.29	DOWN	ESI(−)
Citric acid	$C_6H_8O_7$	[M-H]⁻	191.01882	1.79	0.78	DOWN	ESI(−)
Threonic Acid	$C_4H_8O_5$	[(M-H)-H2O]⁻	117.01812	5.54	1.09	DOWN	ESI(−)
Gamma-glutamyl-ornithine	$C_{10}H_{19}N_3O_5$	[M-H]⁻	260.12458	0.20	0.50	DOWN	ESI(−)
Hydriodic acid	HI	[M-H]⁻	126.90385	4.74	0.46	DOWN	ESI(−)
Leucyl-threonine	$C_{10}H_{20}N_2O_4$	[M-H]⁻	231.13433	0.62	1.61	DOWN	ESI(−)
Val Pro Ala	$C_{13}H_{23}N_3O_4$	[(M-H)-H2O]⁻	266.15091	1.71	8.11	DOWN	ESI(−)
Guanosine 2',3'-cyclic phosphate	$C_{10}H_{12}N_5O_7P$	[M-H]⁻	344.03943	0.35	1.85	DOWN	ESI(−)
N2-Acetyl-ornithine	$C_7H_{14}N_2O_3$	[M-H]⁻	173.09211	2.83	0.98	DOWN	ESI(−)
Glu Ala	$C_8H_{14}N_2O_5$	[(M-H)-H2O]⁻	199.07150	1.86	1.41	DOWN	ESI(−)
Val Ala Arg	$C_{14}H_{28}N_6O_4$	[M-H]⁻	343.20940	0.09	2.25	DOWN	ESI(−)
Gly Pro Val	$C_{12}H_{21}N_3O_4$	[M-H]⁻	270.14542	0.16	1.65	DOWN	ESI(−)
Glutaminylleucine	$C_{11}H_{21}N_3O_4$	[M-H]⁻	258.14549	0.44	2.84	DOWN	ESI(−)
2-Picolinic acid	$C_6H_5NO_2$	[M-H]⁻	122.02357	5.10	0.74	DOWN	ESI(−)
Leucyl-Alanine	$C_9H_{18}N_2O_3$	[M-H]⁻	201.12361	1.46	2.09	DOWN	ESI(−)
N6-acetyl-lysine	$C_8H_{16}N_2O_3$	[M-H]⁻	187.10778	2.52	0.90	DOWN	ESI(−)
Thymidine	$C_{10}H_{14}N_2O_5$	[M-H]⁻	241.08237	0.25	2.09	DOWN	ESI(−)
2-(2-Carboxy-4-methylthiazol-5-yl)ethyl phosphate	$C_7H_{10}NO_6PS$	[M-H]⁻	265.98834	1.74	1.13	DOWN	ESI(−)
Inosine	$C_{10}H_{12}N_4O_5$	[M-H]⁻	267.07295	0.05	2.37	DOWN	ESI(−)
Threonic acid	$C_4H_8O_5$	[M-H]⁻	135.02875	4.31	0.62	UP	ESI(−)
Asparaginyl-Isoleucine	$C_{10}H_{19}N_3O_4$	[M-H]⁻	244.12981	0.32	2.72	DOWN	ESI(−)
Glutamylglycine	$C_7H_{12}N_2O_5$	[(M-H)-H2O]⁻	185.05584	2.06	0.50	DOWN	ESI(−)
2'-Deoxyuridine	$C_9H_{12}N_2O_5$	[M-H]⁻	227.06665	0.59	0.98	DOWN	ESI(−)
Uridine 5'-diphosphate	$C_9H_{14}N_2O_{12}P_2$	[(M-H)-H2O]⁻	384.98185	5.05	7.14	DOWN	ESI (−)
Malic acid	$C_4H_6O_5$	[(M-H)-H2O]⁻	115.00247	5.67	0.50	UP	ESI (−)
O-Acetylserine	$C_{13}H_{21}N_2O_7PS$	[M+2H]⁺	191.04840	1.18	0.70	DOWN	ESI (+)

1-Stearoylglycerophosphoglycerol	$C_{24}H_{49}O_9P$	$[M+H]^+$	513.31916	0.14	6.18	DOWN	ESI (+)
N5-Acetyl-N2-gamma-glutamyl-L-ornithine	$C_{12}H_{21}N_3O_6$	$[(M+H)-H_2O]^+$	286.13940	3.09	0.50	DOWN	ESI (+)
Guanosine 2',3'-cyclic phosphate	$C_{10}H_{12}N_5O_7P$	$[M+H]^+$	346.05428	2.79	1.80	DOWN	ESI (+)
Adenosine 2'-phosphate	$C_{10}H_{14}N_5O_7P$	$[M+H]^+$	348.06987	2.94	0.86	DOWN	ESI (+)
N5-Hydroxy-ornithine	$C_5H_{12}N_2O_3$	$[(M+H)-H_2O]^+$	131.08167	2.85	0.47	UP	ESI (+)
Guanine	$C_5H_5N_5O$	$[M+H]^+$	152.05664	4.14	0.78	UP	ESI (+)
Adenosine 2',3'-cyclic phosphate	$C_{10}H_{12}N_5O_6P$	$[M+H]^+$	330.05936	2.94	1.98	DOWN	ESI (+)
Pyridoxine	$C_8H_{11}NO_3$	$[M+H]^+$	170.08117	3.14	0.54	UP	ESI (+)
Pantothenate	$C_9H_{17}NO_5$	$[M+H]^+$	220.11785	2.90	2.94	DOWN	ESI (+)
6-Deoxy-glucose	$C_6H_{12}O_5$	$[M+H]^+$	165.07572	3.43	5.37	UP	ESI (+)
Asn Arg	$C_{10}H_{20}N_6O_4$	$[(M+H)-H_2O]^+$	271.15292	3.93	1.83	DOWN	ESI (+)
Ethanolamine	C_2H_7NO	$[M+H]^+$	62.060602	0.35	0.47	DOWN	ESI (+)
Indoleacetic acid	$C_{10}H_9NO_2$	$[M+H]^+$	176.07052	3.56	5.08	DOWN	ESI (+)
5-Methyltetrahydropteroyltri-glutamate	$C_{25}H_{36}N_8O_{12}$	$[M+2H]^+$	321.13042	0.10	5.37	UP	ESI (+)
Glutamine	$C_5H_{10}N_2O_3$	$[(M+H)-H_2O]^+$	275.13463	3.03	1.05	DOWN	ESI (+)
Glutamate	$C_5H_9NO_4$	$[M+H]^+$	148.06038	4.01	0.47	DOWN	ESI (+)
Malyl N-acetyl-alpha-glucosaminide	$C_{12}H_{19}NO_{10}$	$[M+H]^+$	338.10763	3.21	5.36	UP	ESI (+)
2-Hydroxy-2-methyl-butanenitrile	C_5H_9NO	$[(2M+H)-H_2O]^+$	181.13370	2.18	3.92	DOWN	ESI (+)
N-Acetyl-hexosamine	$C_8H_{15}NO_6$	$[(M+H)-H_2O]^+$	204.08663	2.73	0.48	DOWN	ESI (+)
Leu Phe	$C_{15}H_{22}N_2O_3$	$[(M+2Na)-H]^+$	323.13589	3.54	5.33	UP	ESI (+)
7-Methyladenine	$C_6H_7N_5$	$[2M+H]^+$	299.14852	1.42	5.36	UP	ESI (+)
gamma-Glutamyl-2-aminobutyrate	$C_9H_{16}N_2O_5$	$[(M+H)-H_2O]^+$	215.10237	3.74	0.51	UP	ESI (+)
Acetyl-2-hydroxy-butanoic acid	$C_6H_{10}O_4$	$[(M+H)-H_2O]^+$	129.05471	3.44	7.16	UP	ESI (+)
Pyroglutamyl valine	$C_{10}H_{16}N_2O_4$	$[M+H]^+$	229.11806	3.33	0.50	UP	ESI (+)
Hydroxyacetone	$C_3H_6O_2$	$[M+K]^+$	113.00012	3.17	0.47	DOWN	ESI (+)
Met-Val-OH	$C_{15}H_{20}N_2O_6S$	$[(M+H)-H_2O]^+$	339.10188	1.24	5.36	UP	ESI (+)
Pantothenamide	$C_9H_{18}N_2O_4$	$[M+H]^+$	219.13376	3.24	0.50	DOWN	ESI (+)
4-Aminobutanoate	$C_4H_9NO_2$	$[(M+H)-H_2O]^+$	86.060484	1.11	9.44	DOWN	ESI (+)
N-Acetylproline	$C_7H_{11}NO_3$	$[M+H]^+$	158.08118	3.34	0.47	UP	ESI (+)
N-palmitoyl tyrosine	$C_{25}H_{41}NO_4$	$[(M+H)+2K]^+$	166.07902	3.42	5.36	UP	ESI (+)
2-Oxoarginine	$C_6H_{11}N_3O_3$	$[M+H]^+$	174.08727	3.35	0.54	UP	ESI (+)
Pantoate	$C_6H_{12}O_4$	$[(M+2Na)-H]^+$	193.04429	5.04	0.70	DOWN	ESI (+)
5-Hydroxykynurenamine	$C_9H_{12}N_2O_2$	$[M+H]^+$	181.09708	3.40	2.53	DOWN	ESI (+)
Aminoacetone	C_3H_7NO	$[(M+H)-H_2O]^+$	56.05009	1.30	12.77	DOWN	ESI (+)
N-Acetylcadaverine	$C_7H_{16}N_2O$	$[M+H]^+$	145.13358	3.79	0.87	DOWN	ESI (+)
Triethylamine	$C_6H_{15}N$	$[M+H]^+$	102.12796	2.77	10.02	DOWN	ESI (+)
Asn-Arg	$C_{10}H_{20}N_6O_4$	$[(M+H)-H_2O]^+$	271.15176	0.41	0.51	DOWN	ESI (+)
Imidazolepropionic acid	$C_6H_8N_2O_2$	$[(M+H)-H_2O]^+$	123.05543	3.30	1.17	DOWN	ESI (+)
N-Acetylputrescine	$C_6H_{14}N_2O$	$[(M+H)-H_2O]^+$	113.10756	2.72	0.47	UP	ESI (+)
Homoserine lactone	$C_4H_7NO_2$	$[(M+H)-H_2O]^+$	84.044834	1.13	0.47	DOWN	ESI (+)
Adenine	$C_5H_5N_5$	$[M+H]^+$	136.06174	4.20	3.70	DOWN	ESI (+)
Cytosine	$C_4H_5N_3O$	$[M+H]^+$	112.05080	2.43	0.82	DOWN	ESI (+)

Cadaverine	$C_5H_{14}N_2$	$[M+H]^+$	103.12328	2.24	0.43	UP	ESI (+)
LysoPA(i-19:0/0:0)	$C_{22}H_{45}O_7P$	$[M+2H]^+$	227.15219	3.40	2.23	UP	ESI (+)
3-Butyn-1-al	C_4H_4O	$[M+H]^+$	69.034038	0.12	5.37	UP	ESI (+)
Thiamine monophos-phate	$C_{12}H_{17}N_4O_4PS$	$[M+2H]^+$	173.04204	6.84	0.47	DOWN	ESI (+)
Homophenylalanine	$C_{10}H_{13}NO_2$	$[M+H]^+$	180.10186	3.27	3.70	DOWN	ESI (+)
Putrescine	$C_4H_{12}N2$	$[M+H]^+$	89.10775	1.34	0.43	UP	ESI (+)
N-Carbamoylputrescine	$C_5H_{13}N_3O$	$[(M+H)-H_2O]^+$	114.10284	2.41	0.47	DOWN	ESI (+)
5-amino-pentanoic acid	$C_5H_{11}NO_2$	$[(M+H)-H_2O]^+$	100.07600	2.20	0.50	DOWN	ESI (+)
gamma-Glutamyl-beta-aminopropiononitrile	$C_8H_{13}N_3O_3$	$[M+Na]^+$	222.08471	3.36	0.70	UP	ESI (+)
4-Hydroxyphenylglycine	$C_8H_9NO_3$	$[M+H]^+$	168.06547	3.46	0.90	UP	ESI (+)
Uracil	$C_4H_4N_2O_2$	$[M+H]^+$	113.03476	2.99	2.05	DOWN	ESI (+)
Phenylpyruvic acid	$C_9H_8O_3$	$[M+H]^+$	165.05456	3.59	8.01	DOWN	ESI (+)
Guanosine	$C_{10}H_{13}N_5O_5$	$[M+H]^+$	284.09845	3.64	2.43	DOWN	ESI (+)
Valine	$C_5H_{11}NO_2$	$[M+H]^+$	118.08642	3.18	0.47	DOWN	ESI (+)
3-Cyanoalanine	$C_4H_6N_2O_2$	$[(2M+H)-H_2O]^+$	211.08268	2.08	0.50	DOWN	ESI (+)
D-Alanyl-D-alanine	$C_6H_{12}N_2O_3$	$[M+H]^+$	161.09206	3.37	0.50	DOWN	ESI (+)
Threonine	$C_4H_9NO_3$	$[(M+H)-H_2O]^+$	102.05526	2.26	0.47	DOWN	ESI (+)
5-Aminopentanamide	$C_5H_{12}N_2O$	$[M+H]^+$	117.10239	3.27	0.49	DOWN	ESI (+)
Alanyl-Leucine	$C_9H_{18}N_2O_3$	$[M+H]^+$	203.13897	2.87	2.75	DOWN	ESI (+)
Lysine	$C_6H_{14}N_2O_2$	$[M+H]^+$	147.11277	3.89	0.43	DOWN	ESI (+)
N-Butyryl-homoserine lactone	$C_8H_{11}NO$	$[(M+H)-H_2O]^+$	136.07569	3.96	3.74	DOWN	ESI (+)
Biotin sulfoxide	$C_{10}H_{16}N_2O_4S$	$[M+H]^+$	261.08995	3.62	2.09	UP	ESI (+)
Thymine	$C_5H_6N_2O_2$	$[M+H]^+$	127.05031	3.44	2.75	DOWN	ESI (+)
Arginine	$C_6H_{14}N_4O_2$	$[(M+H)-H_2O]^+$	157.10836	3.56	0.70	UP	ESI (+)
N2-Succinyl-ornithine	$C_9H_{16}N_2O_5$	$[(M+H)-H_2O]^+$	197.09200	3.05	3.30	DOWN	ESI (+)
Tyramine	$C_8H_{11}NO$	$[(M+H)-H_2O]^+$	120.08091	3.34	2.94	DOWN	ESI (+)
beta-Alanyl-arginine	$C_9H_{19}N_5O_3$	$[(M+H)-H_2O]^+$	228.14533	3.07	0.98	DOWN	ESI (+)
Betaine	$C_5H_{11}NO_2$	$[(2M+H)-H_2O]^+$	217.15451	3.26	2.39	DOWN	ESI (+)
Ornithine	$C_5H_{12}N_2O_2$	$[(M+H)-H_2O]^+$	115.08673	3.41	0.47	DOWN	ESI (+)
N-gamma-Glutamylglu-tamine	$C_{10}H_{17}N_3O_6$	$[(M+H)-H_2O]^+$	258.10816	3.20	0.87	DOWN	ESI (+)

*RT: retention time.

Table S4. Antimicrobial activity of EOs on *Salmonella enterica* serovar Enteritidis ATCC 13076.

**Code**	**Plant species**	***Salmonella* Enteritidis ATCC 13076**	
		**MIC_{50} (mg/mL)**	**MBC (mg/mL)**
SA	*Steiractinia aspera* Cuatrec.	> 1.5	> 1.5
TD-I	*Turnera diffusa* Willd	> 1.5	> 1.5
LOP	*Lippia origanoides* H.B.K quimiotipo felandreno	> 1.5	> 1.5

**Code**	**Plant species**	***Salmonella* Enteritidis ATCC 13076**	
		**MIC_{50} (mg/mL)**	**MBC (mg/mL)**
CM-I	*Calycolpus moritzianus* Burret	> 1.5	> 1.5
PA	*Piper aduncum* Lam	> 1.5	> 1.5
EQ	*Elaphandra quinquenervis* H.Rob	> 1.5	> 1.5
HD	*Hyptis dilatate* Benth	> 1.5	> 1.5
LOC	*L. origanoides* H.B.K quimiotipo carvacrol	0.75 ± 0.0041	1.5
LOCpT	*L. origanoides* H.B.K quimiotipo β-cariofileno-timol	> 1.5	> 1.5
LOT-I	*L. origanoides* H.B.K quimiotipo timol	> 1.5	> 1.5
TD-II	*T. diffusa* Willd	> 1.5	> 1.5
SV	*Satureja viminea* (L.) Kuntze	> 1.5	> 1.5
PS	*Psidium sartorianum* (O.Berg) Nied	> 1.5	> 1.5
VC	*Varronia curassavica* Jacq.	> 1.5	> 1.5
OB	*Ocimum basilicum* L.	> 1.5	> 1.5
CM-II	*C. moritzianus* Burret	> 1.5	> 1.5
TD-III	*T. diffusa* Willd	> 1.5	> 1.5
LOTC	*L. origanoides* H.B.K quimiotipo timol-*p*-cimeno	0.37 ± 0.0070	0.75
LOT-II	*L. origanoides* H.B.K quimiotipo timol	0.37 ± 0.0043	0.75
LM	*L. micromera* Schauer	> 1.5	> 1.5

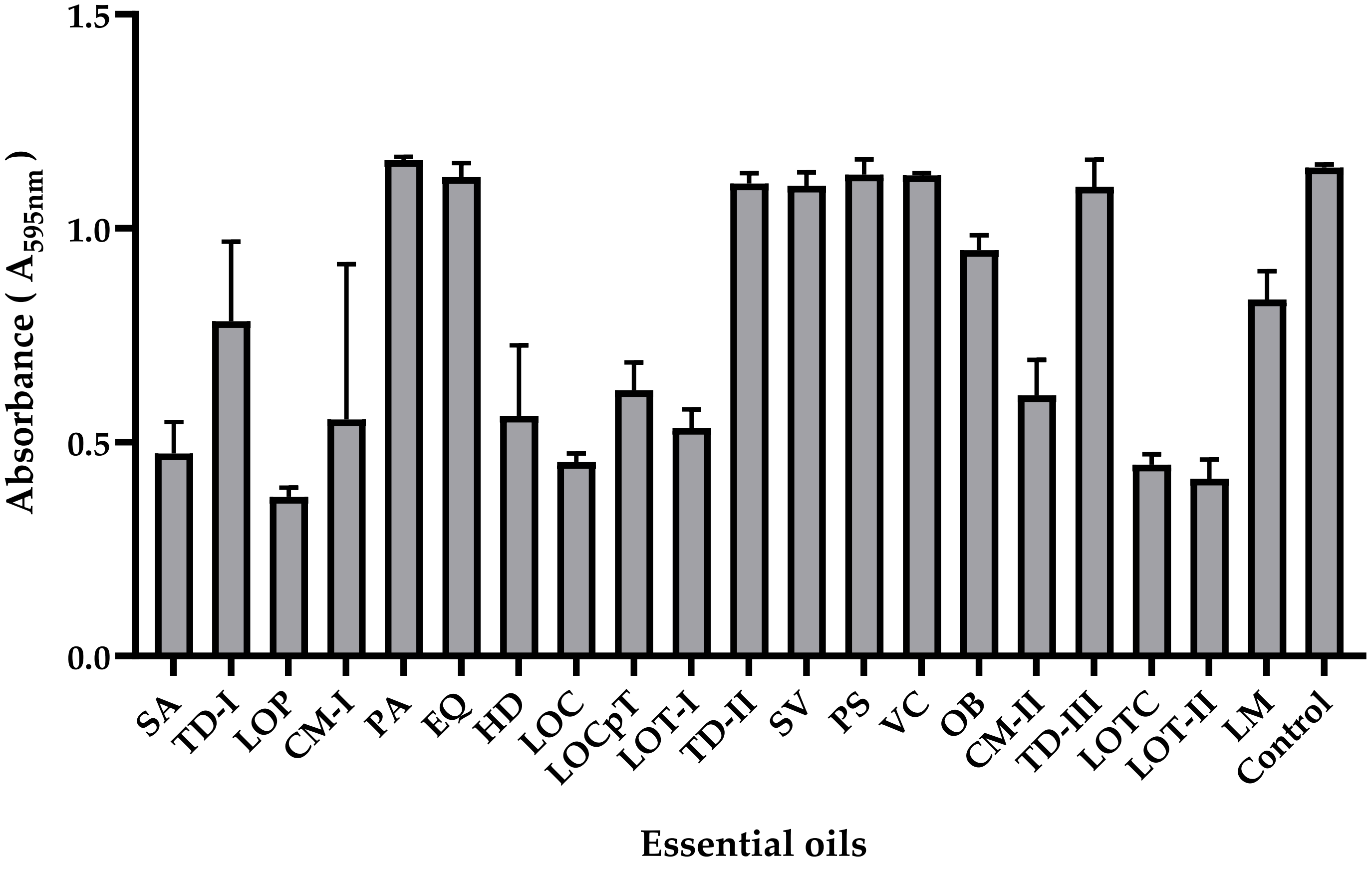

Figure S1. Anti-biofilm activity of different essential oils on *Salmonella enterica* serovar Enteritidis ATCC 13076. The concentrations evaluated were as follows: EOs SA, TD-I, LOP, CM-I, PA, EQ, HD, TD-II, SV, PS, VC, OB, CM-II, and TD-III at 1.5 mg/mL; LOCpT and LM at 0.75 mg/mL; LOC and LOT-I at 0.18 mg/mL; LOTC and LOT-II at 0.13 mg/mL.
